# Androgen receptor splice variant 7 expression levels distinguish *AR*-mutated from nonmutated metastatic castration-resistant prostate cancers

**DOI:** 10.1172/JCI198193

**Published:** 2026-04-01

**Authors:** Alec Paschalis, Ines Figueiredo, Denisa Bogdan, Arian Lundberg, Rita Santos, Bora Gurel, Tarek Taha, Ossian Longoria, Ana Ferreira, Claudia Bertan, Nicholas Brittain, Ryan Nelson, Laura Walker, Antje Neeb, Jonathan Welti, Wei Yuan, Costas Mitsopoulos, Stephen R. Plymate, Michael C. Haffner, Adam G. Sowalsky, Suzanne Carreira, Adam Sharp, Luke Gaughan, Johann de Bono

**Affiliations:** 1 The Institute of Cancer Research, London, United Kingdom.; 2 The Royal Marsden Hospital, London, United Kingdom.; 3 Newcastle University Centre for Cancer, United Kingdom.; 4 Department of Medicine, University of Washington School of Medicine and VAPSHCS-GRECC, Seattle, Washington, USA.; 5 Divisions of Human Biology and Clinical Research, Fred Hutchinson Cancer Center, Seattle, Washington, USA.; 6 Department of Laboratory Medicine and Pathology, University of Washington, Seattle, Washington, USA.; 7 Genitourinary Malignancies Branch, National Cancer Institute, Bethesda, Maryland, USA.

**Keywords:** Clinical Research, Oncology, Prostate cancer

## Abstract

New androgen receptor (AR) pathway inhibitors (ARPIs) in clinical development, including AR degraders and CYP11A inhibitors, largely target ligand-dependent AR activation and have reported antitumor activity in metastatic castration-resistant prostate cancer (mCRPC) resistant to established ARPIs, predominately against tumors with *AR* mutations. We hypothesized that *AR*-mutated mCRPC exhibits lower AR splice variant 7 (AR-V7) expression and remains full-length–AR (FL-AR) driven, explaining, in part, the antitumor activity of these AR ligand–binding domain (LBD) targeting drugs. The data herein demonstrate that mCRPC tissue biopsies with detectable *AR* mutations express significantly lower levels of AR-V7 protein and associate with better overall survival and enhanced sensitivity to ARPIs. This is independent of differences in the total number of global splicing events but may be related to differences in splicing factor expression between *AR*-mutated and nonmutated mCRPC. In conclusion, *AR*-mutated mCRPC frequently exhibits low AR-V7 expression, arguably explaining the enhanced sensitivity to ARPIs observed in these cancers. Consequently, *AR* mutation status may serve as a biomarker to predict response to AR-directed therapies.

## Abstract 2

Advanced prostate cancers with detectable mutations of the androgen receptor (AR) frequently have low levels of AR-V7 and exhibit enhanced sensitivity to AR pathway inhibitors.

## Introduction

Prostate cancer (PC) is one of the most common cancers in men and is a leading cause of male cancer death globally. Deregulated androgen receptor (AR) signaling is a key driver of PC development and progression and remains a major therapeutic target in advanced disease, with AR pathway inhibitors (ARPIs) having improved clinical outcomes from PC (1, 2). However, all advanced PCs eventually become resistant to ARPIs, such as abiraterone, enzalutamide, apalutamide, and darolutamide, leading to fatal disease progression. Resistance to ARPIs can develop due to the emergence of constitutively active alternatively spliced variants of the AR (AR-Vs) that lack the regulatory AR ligand-binding domain (LBD), which is the target of currently approved and new ARPIs in clinical development, including AR degraders and CYP11A inhibitors (3–5). Of the many AR-Vs reported, AR splice variant 7 (AR-V7) is the most prevalent and associates with resistance to AR targeting therapies and worse prognosis (4, 6).

The development of new ARPIs, as well as predictive biomarkers to identify patients most likely to benefit from these therapies, remain areas of urgent unmet clinical need. Recent clinical trials of novel AR-degraders and CYP11A inhibitors have reported antitumor activity in heavily pretreated patients with metastatic castration-resistant prostate cancer (mCRPC), specifically in tumors harboring *AR* mutations (7–9). In the recent Phase I trial of ODM-208, an oral nonsteroidal selective inhibitor of CYP11A1, 38.1% of evaluable patients achieved a PSA decline of greater than or equal to 50% (PSA50). However the PSA50 rate among those with detectable mutations of the *AR* LBD was 73.3% (7). Similarly, in the Phase I/II trial of ARV-110, an oral proteolysis-targeting chimera (PROTAC) protein degrader targeting full-length AR (FL-AR) and clinically relevant mutants, the PSA50 rate was found to be 10% among patients without detectable *AR* mutations, but 46% in patients with AR T878X/H875Y–positive tumors (8). Furthermore, while the early phase clinical trial of BMS-986365, an AR degrader and antagonist, has reported efficacy in patients without *AR* mutations, PSA50 rates were again reported to be higher in patients with detectable *AR* mutation; 27% in patients with nonmutated *AR* LBD and 55% in patients with *AR* LBD mutations (9).

We hypothesized that these clinical trial data indicate that PCs with *AR* mutations have limited *AR* splicing and remain FL-AR driven, arguably explaining the antitumor activity observed with these AR LBD-targeting drugs in clinical development. Herein, we report that mutations of the *AR* are identifiable in 14% of clinical mCRPC biopsies, with *AR*-mutated mCRPC biopsies exhibiting significantly lower AR-V7 protein levels. Consistent with this, patients with detectable *AR* mutations in their mCRPC biopsies demonstrate greater sensitivity to ARPI therapy and improved overall survival (OS) from diagnosis, indicating that *AR* mutation status may be both prognostic and a predictive biomarker of response to ARPI therapy.

## Results

### Patients with detectable AR mutation in their mCRPC tissue biopsies derive greater benefit from AR-directed therapy.

**Figure 1:**
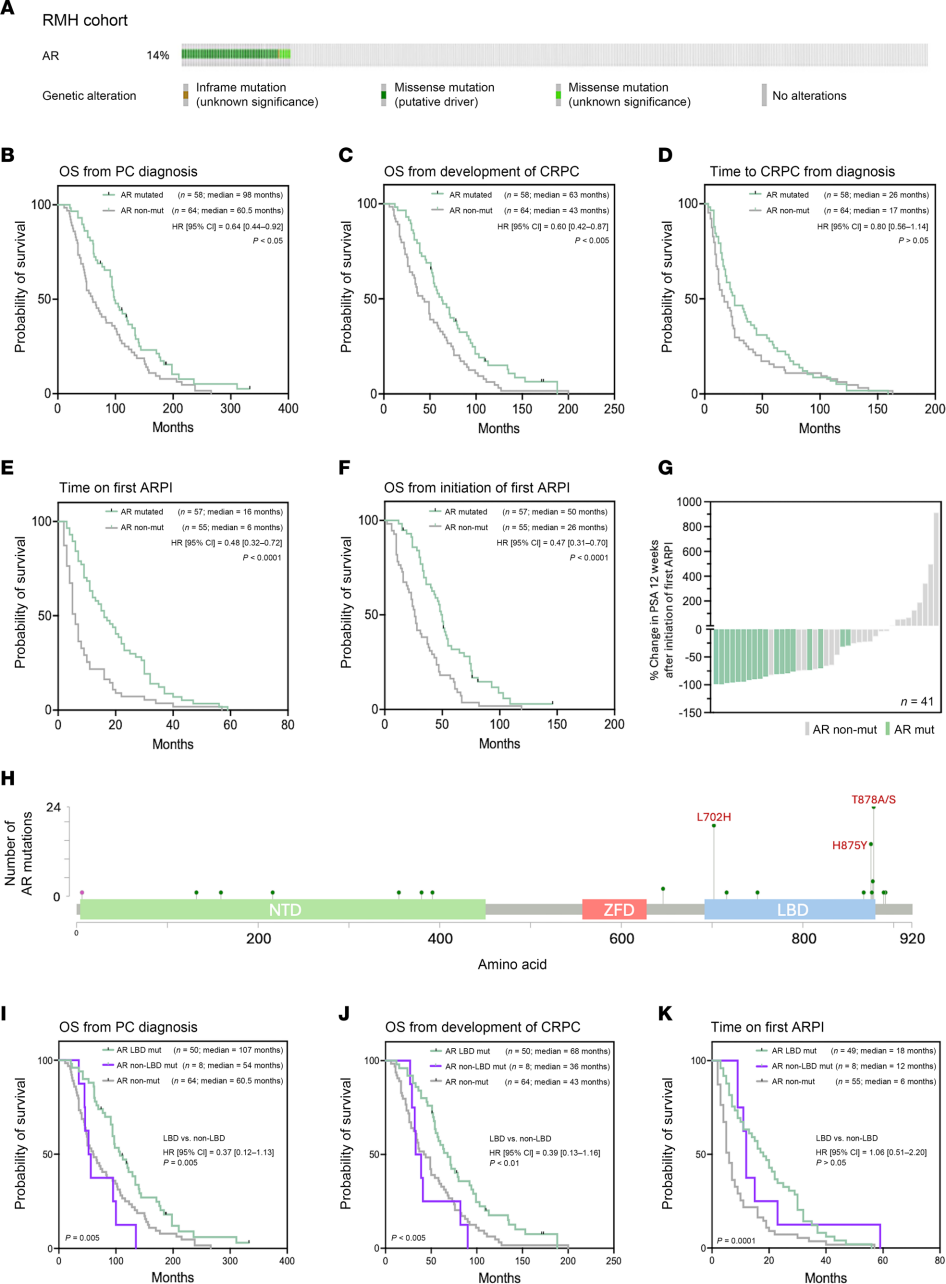
*AR* mutation is associated with a better clinical outcome in advanced PC. (**A**) Oncoprint diagram illustrating the frequency and type of *AR* alteration in a cohort of 475 metastatic CRPC (mCRPC) biopsies with available next-generation sequencing (NGS) data (RMH clinical cohort). (**B**–**F**) Kaplan-Meier curves comparing clinical outcomes between patients with and without detectable *AR* mutation (RMH clinical cohort). Detectable *AR* mutation in mCRPC tissue biopsy associated with improved overall survival from prostate cancer (PC) diagnosis (**B**; *n* = 122, *P* < 0.05; Log-rank test) and development of CRPC (**C**; *n* = 122, *P* < 0.005; Log-rank test), with a longer time to development of CRPC (**D**; *n* = 122, *P* > 0.05; Log-rank test). Detectable *AR* mutation also associated with a longer time on first AR pathway inhibitor (ARPI) (**E**; *n* = 112, *P* < 0.0001; Log-rank test) and overall survival from initiation of first ARPI (**F**; *n* = 112, *P* < 0.0001; Log-rank test). (**G**) Waterfall plot demonstrating percentage (%) PSA change from baseline 12 weeks after commencement of first ARPI (Abiraterone/Enzalutamide). All patients received ARPI in the CRPC setting. Each bar represents an individual patient within the RMH clinical cohort with sufficient clinical data for evaluation. Patients with a detectable *AR* mutation shown in green. Those without a detectable *AR* mutation shown in grey. (**H**) Lollipop graph illustrating the location and frequency of *AR* mutation identified within the evaluated RMH clinical cohort of mCRPC patient biopsies, with the most frequent mutations highlighted in red. (**I**–**K**) Kaplan-Meier curves comparing clinical outcomes between patients with *AR* LBD mutations, *AR* non-LBD mutations, or without detectable *AR* mutation (RMH clinical cohort). Overall, *AR* LBD mutation associated with significantly improved overall survival from diagnosis (**I**; *n* = 122, *P* = 0.005; Log-rank test), overall survival from development of CRPC (**J**; *n* = 122, *P* < 0.005; Log-rank test), and time on first ARPI (**K**; *n* = 112, *P* = 0.0001; Log-rank test), than those without detectable *AR* mutations. However, this was not the case for patients with non-LBD *AR* mutations. NTD, AR n-terminal domain; ZFD, AR zinc-finger domain; LBD, AR ligand binding domain.

In keeping with previous reports (10, 11), interrogation of targeted sequencing data from 475 patients with mCRPC treated at the Royal Marsden Hospital (RMH) identified 68 patients (14.3%) with *AR* mutations (Figure 1A and Supplemental Table 1; supplemental material available online with this article; https://doi.org/10.1172/JCI198193DS1). Of these 68 patients, 58 had sufficient previously collected formalin-fixed, paraffin-embedded (FFPE) mCRPC biopsy tissue and clinical data available for further assessment and were compared against a matched cohort of patients with mCRPC without detectable *AR* mutations, with sufficient tissue and clinical data available for comparison (RMH clinical cohort; [*n* = 64]; Supplemental Figure 1 and Supplemental Table 2). Retrospective evaluation of these patients’ medical records revealed that patients with detectable *AR* mutations in their mCRPC tissue biopsies had significantly better OS from both diagnosis (98 months [*n* = 58] versus 60.5 months [*n* = 64]; hazard ratio 0.64; 95% confidence interval 0.44–0.92; *P* < 0.05) and development of CRPC (63.0 months [*n* = 58] versus 43.0 months [*n* = 64]; hazard ratio 0.60; 95% confidence interval 0.42–0.87; *P* < 0.005) compared with those without detectable *AR* mutations (Figure 1, B and C), with a longer time to development of CRPC (26 months [*n* = 58] versus 17 months [*n* = 64]; hazard ratio 0.80; 95% confidence interval 0.56–1.15; *P* > 0.05) (Figure 1D). Furthermore, these patients achieved a longer time on AR-directed therapy (16.0 months [*n* = 57] versus 6.0 months [*n* = 55]; hazard ratio 0.48; 95% confidence interval 0.32–0.72; *P* < 0.0001) and had a better OS from initiation of their first ARPI (50.0 months [*n* = 57] versus 26.0 months [*n* = 55]; hazard ratio 0.47; 95% confidence interval 0.31–0.70; *P* < 0.0001) (Figure 1, E and F). Similarly, among patients with available PSA response data (*n* = 41), those with mCRPC tumors with detectable *AR* mutations exhibited greater reductions in PSA levels 12 weeks after the initiation of their first ARPI therapy than those with tumors without detectable *AR* mutation (Figure 1G).

Next, to investigate the clinical relevance of specific *AR* mutations, identified mutations were subdivided into those involving the *AR* LBD and those involving other regions of the *AR* gene, with clinical outcomes compared between these 2 groups. Of the 58 patients with detectable *AR* mutations evaluated, 43 (74%) had isolated mutations of the *AR* gene while 15 (26%) had 2 or more cooccurring mutations (Supplemental Table 1). Interestingly, among patients with more than 1 mutation of *AR*, these were typically both mutations of the *AR* LBD (*n* = 12; 80%), with only 3 (20%) patients having separate mutations in both the LBD and non-LBD regions of the *AR* gene. For the purposes of these analyses therefore, all patients with at least one detectable mutation of the *AR* LBD were considered *AR* LBD mutant, except for 1 patient who, despite having a tumor content of 95%, had an LBD mutation allele frequency of only 7%, with a non-LBD mutation allele frequency of 99%, so was classified as non-LBD mutant (Supplemental Table 1).

Overall, 50 patients (86%) had 1 or more mutations within the *AR* LBD coding region identifiable in their mCRPC tissue biopsy, with the well characterized activating amino acid point mutations T878A/S (*n* = 22; 30% of *AR* mutations), L702H (*n* = 19; 26%), and H875Y (*n* = 13; 18%) that increase AR ligand promiscuity occurring most frequently (Figure 1H). Mutations of the *AR* LBD were associated with better OS from both diagnosis (*P* = 0.005; Log-rank) and development of CRPC (*P* < 0.005; Log-rank), with this being independent of AR-V7 status (Supplemental Figure 2). However, there was no significant difference in clinical outcomes between patients with non-LBD *AR* mutations and those without detectable *AR* mutations (OS from diagnosis: non-LBD mutant 54 months [*n* = 8] versus nonmutant 60.5 months [*n* = 64]; Hazard ratio 1.32; 95% confidence interval 0.58–3.02; *P* > 0.05; and OS from development of CRPC: non-LBD mutant 36 months [*n* = 8] versus nonmutant 43 months [*n* = 64]; Hazard ratio 1.20; 95% confidence interval 0.54–2.64; *P* > 0.05; Figure 1, I and J). Similarly, patients with detectable *AR* LBD mutations in their mCRPC biopsies remained on ARPI therapy the longest (*P* = 0.0001; Log-rank; Figure 1K). Taken together, these data suggest that, while mutations of the *AR* LBD are associated with improved clinical outcomes, mCRPC with *AR* mutations in other regions has similar clinical outcomes to mCRPC without detectable *AR* mutation.

### AR LBD mutated mCRPC tissue biopsies exhibit lower levels of AR-V7 protein.

We hypothesized that the different clinical outcomes observed with *AR*-mutated mCRPC compared with *AR*-nonmutated mCRPC was at least in part due to differences in AR splice variant expression between these 2 groups. AR-V7 protein levels were therefore evaluated across our RMH clinical cohort (*n* = 122) and compared between patients with (*n* = 58) and without (*n* = 64) detectable *AR* mutations. *AR*-mutated mCRPC biopsies were subsequently further subdivided into those involving the *AR* LBD and those that did not involve the *AR* LBD. Surprisingly, patients with mCRPC with detectable *AR* mutations did not have a history of different exposure to abiraterone (*n* = 26; 45%) versus enzalutamide (*n* = 26; 45%) (Supplemental Table 2).

**Figure 2:**
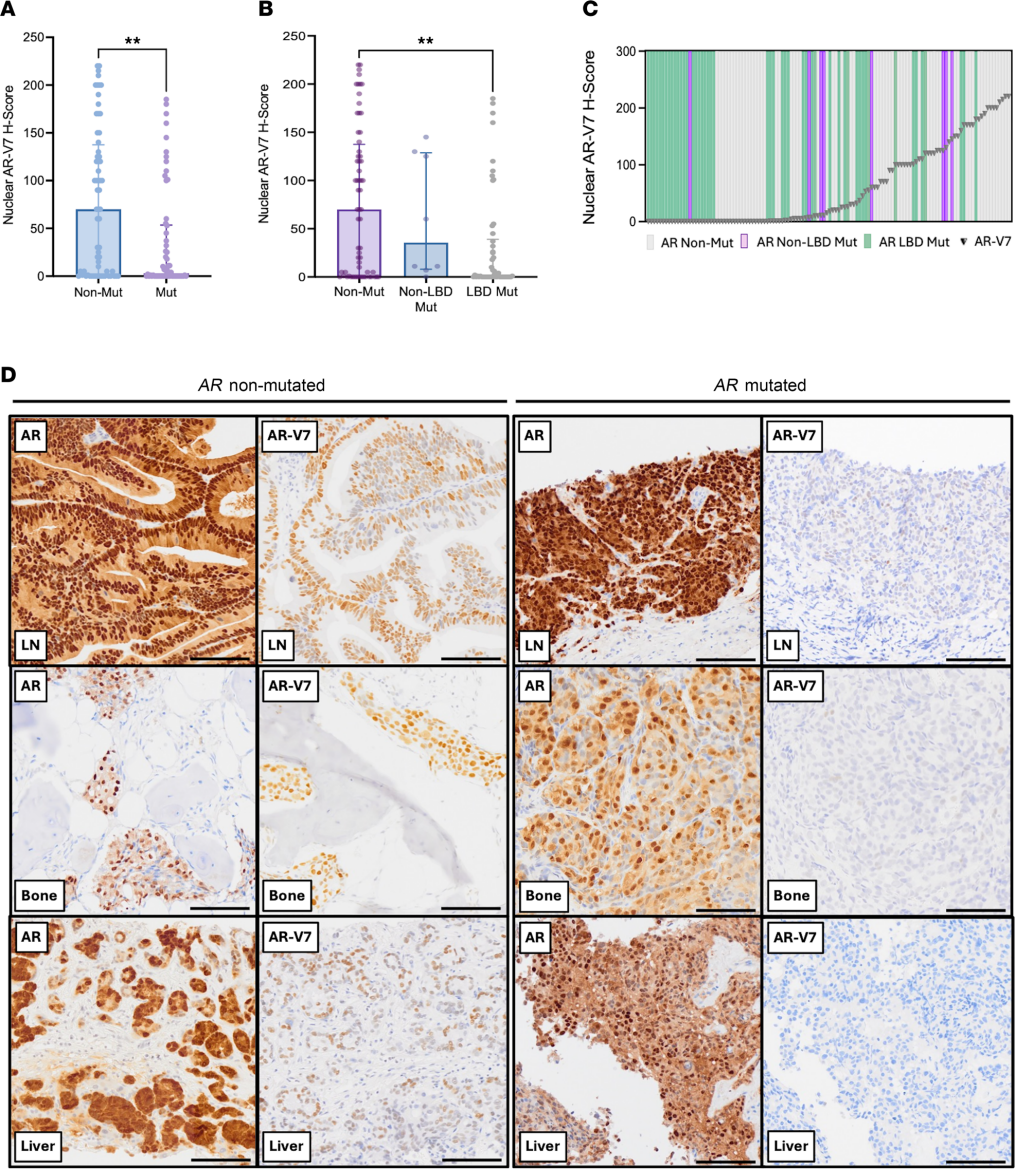
mCRPC patient tissue samples with detectable *AR* mutations exhibit lower levels of AR-V7 protein. (**A**) Bar graph of AR-V7 protein levels (IHC H-Score) in mCRPC patient biopsies with and without detectable *AR* mutations (RMH clinical cohort). Median H-Score and interquartile range shown. Statistical significance determined by Mann-Whitney test (*P < 0.05, **P < 0.01, ***P < 0.001). (**B**) Bar graph of AR-V7 protein levels (IHC H-Score) in mCRPC patient biopsies with LBD *AR* mutations, non-LBD *AR* mutations, or no detectable *AR* mutations (RMH clinical cohort). Median H-Score and interquartile range shown. Statistical significance determined by Kruskal-Wallis with Dunn’s multiple-comparisons test (*P < 0.05, **P < 0.01, ***P < 0.001). (**C**) Amalgamation of AR-V7 IHC data with *AR* mutation status (targeted sequencing panel) for each individual patient within the RMH clinical cohort (*n* = 122). Green bars indicate patients with detectable *AR* LBD mutations, purple bars indicate patients with non-LBD *AR* mutations, and grey bars indicate patients without detectable *AR* mutations. Grey triangles denote AR-V7 IHC H-Score, determined by analytically validated IHC assay. Results presented in order of increasing AR-V7 H-Score. (**D**) Representative micrographs of IHC analyses for AR NTD (left) and AR-V7 (right) protein levels in matched, same-patient, same-biopsy, mCRPC tissue samples from 6 different patients; 3 of which have a detectable *AR* mutation, 3 of which do not (RMH clinical cohort). Scale bars: 100μm. LN, lymph node; LBD, AR ligand binding domain.

Overall, nuclear AR-V7 protein levels were significantly lower in patients with detectable *AR* mutation (*AR*-mutated median H-score 3.0, IQR [0.0–53.5, *n* = 58] versus *AR*-nonmutated median H-score 70 [0.3–137.5, *n* = 64]; *P* = 0.002; Figure 2, A–C), with representative micrographs of immunohistochemical (IHC) analyses for AR n-terminal domain (NTD) and AR-V7 protein levels in matched, same-patient, same-biopsy, mCRPC tissue samples shown in Figure 2D. Furthermore, subgroup analysis demonstrated a statistically significant difference in AR-V7 expression between *AR*-nonmutated and *AR* LBD-mutated biopsies (median H-score 70 [IQR 0.3–137.5], *n* = 64 versus 1.0 [0.0–39.0], *n* = 50; adjusted *P* = 0.002), with no significant differences observed between the remaining groups. Taken together, these results support the hypothesis that the observed higher antitumor activity of novel ARPIs in clinical development targeting ligand-activated AR signaling in *AR-*mutated mCRPC may be due to lower levels of AR-V7 in these cancers.

### AR-V7 levels in AR-mutated mCRPC tissue biopsies are not dependent on AR amplification alone.

**Figure 3:**
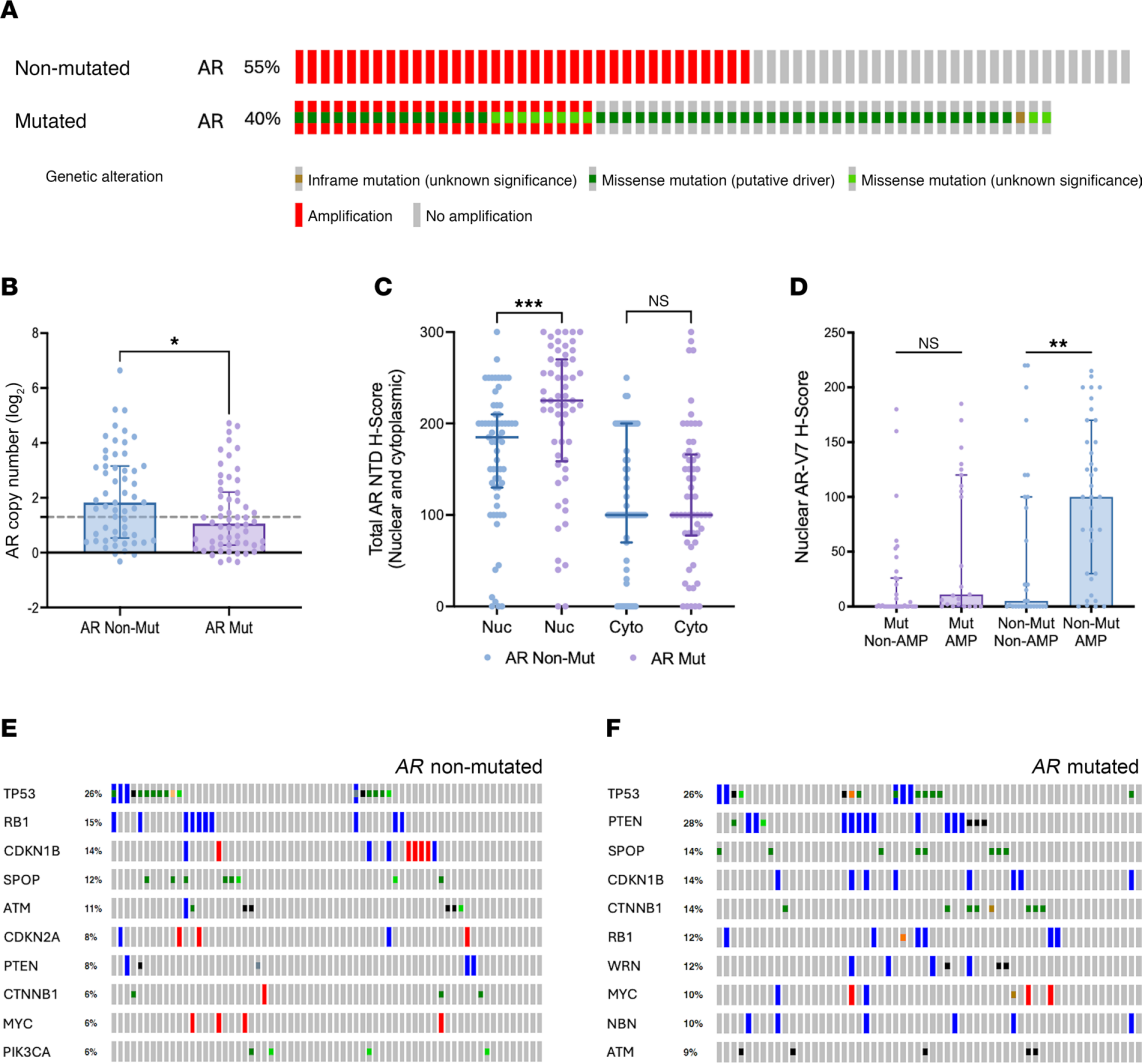
*AR* amplification and AR-V7 expression in *AR*-mutated mCRPC clinical biopsies. (**A**) Oncoprint diagram illustrating the incidence of *AR* gene amplification among evaluated patients with and without detectable *AR* mutations (RMH clinical cohort). (**B**) Bar chart of *AR* copy number in mCRPC patient biopsies with and without detectable *AR* mutations (RMH clinical cohort). Threshold for classification as *AR* gene amplification indicated by dashed grey line. (**C**) Bar chart of nuclear (Nuc) and cytoplasmic (Cyto) AR-n terminal domain (NTD) protein levels (IHC H-Score) in mCRPC patient biopsies with and without detectable *AR* mutations (RMH clinical cohort). (**D**) Bar chart of nuclear AR-V7 protein levels (IHC H-Score) in mCRPC patient biopsies with and without detectable *AR* mutations, subdivided into those with and without *AR* amplification. Median H-Score and interquartile range shown. (**E** and **F**) Oncoprint diagram illustrating the top cooccurring genetic alterations other than *AR* amplification found on targeted sequencing of mCRPC tissue biopsies with and without detectable *AR* mutation. Statistical significance in **B** and **C** determined by Mann-Whitney test. Statistical significance in **D** determined by Kruskal-Wallis test. (*P < 0.05, **P < 0.01, ***P < 0.001). Nuc, Nuclear IHC staining; Cyto, Cytoplasmic IHC staining.

Higher AR-V7 expression has previously been reported to be associated with increased *AR* gene copy number (12). To investigate the relationship between FL-AR and AR-V7 expression in *AR*-mutated and nonmutated mCRPC biopsies, we evaluated *AR* gene copy number and AR total protein expression (using an NTD binding antibody to detect both full-length and splice-variant AR) across our RMH clinical cohort. Overall, *AR* gene amplification, defined as log_2_ (*AR* copy number) greater than 1.3, was identified in 55% (*n* = 35) of evaluated mCRPC biopsies without detectable *AR* mutation and 40% (*n* = 23) of those with detectable *AR* mutation (Figure 3A and Supplemental Table 3), indicating that these are not mutually exclusive genomic alterations. Interestingly, while *AR* copy number was higher in mCRPC biopsies without detectable *AR* mutation (1.1 [0.3–2.2, *n* = 56] versus 1.8 [0.5–3.2, *n* = 57]; *P* < 0.05; Figure 3B), median nuclear AR NTD protein levels were higher in *AR*-mutated mCRPC biopsies (*AR-*mutated median nuclear AR NTD H-Score = 225.0 [158.8–270.0] versus AR-nonmutated median nuclear AR NTD H-Score = 185 [130.0–210.0]; *P* < 0.0005; Figure 3C).

In keeping with previous reports, AR-V7 levels were significantly higher in *AR*-nonmutated mCRPC biopsies with *AR* amplification; *AR-*nonmutated and *AR*-amplified mCRPC had a median nuclear AR-V7 H-Score of 100.0 [30.0–170.0] while *AR-*nonmutated and nonamplified mCRPC had a median nuclear AR-V7 H-Score of 5 [0.0–100.0]; *P_adj_* < 0.005; Figure 3D). However, this was not the case in *AR*-mutated mCRPC biopsies; *AR-*mutated and *AR*-amplified mCRPC had a median nuclear AR-V7 H-Score of 11.0 [0.0–120.0] whereas *AR-*mutated and nonamplified mCRPC had a median nuclear AR-V7 H-Score of 0 [0.0–26.0]; *P_adj_* > 0.05; Figure 3D).

While *AR* amplification was the most common cooccurring genomic alteration detected across both *AR*-mutated and nonmutated mCRPC biopsies, evaluation of available targeting sequencing data revealed 8 of the top 10 cooccurring genomic alterations identified in both *AR*-mutated and nonmutated cancers to be shared across both groups (Figure 3, E and F). Of these, the most notable difference between these 2 groups was the incidence of *PTEN* alterations, which was evident in 28% (*n* = 16) of *AR*-mutated cancers but only 8% (*n* = 5) of *AR*-nonmutated cancers.

Taken together, these data indicate that, while *AR* amplification contributes to increased AR-V7 expression in *AR*-nonmutated mCRPC, this alone may not be sufficient to drive AR-V7 production in *AR*-mutated cancers.

### Meta-analyses reveal differences in the expression of spliceosome-related genes between AR-mutated and nonmutated mCRPC.

**Figure 4:**
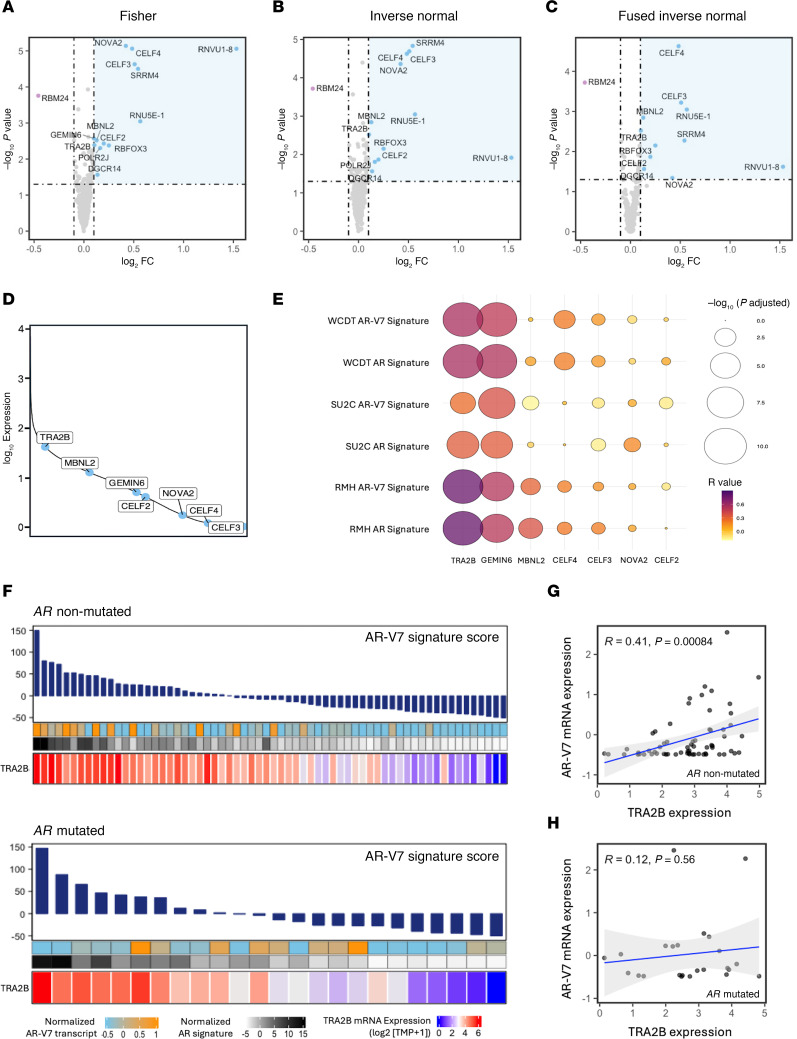
*AR*-mutated biopsies from patients with mCRPC exhibit a distinct splicing factor expression profile. (**A**–**C**) Volcano plots demonstrating the differential gene expression of spliceosome-related genes between mCRPC patient biopsies with and without detectable *AR* mutations (RMH transcriptomic cohort), identified in meta-analyses using 3 statistical methods: (**A**) Fisher, (**B)** Inverse Normal, and (**C**) Fused Inverse Normal. Purple dots represent genes upregulated in patients with detectable *AR* alterations, light blue dots indicate genes upregulated in patients without detectable *AR* alterations, and grey dots denote genes with stable expression between the 2 groups. Dashed horizontal and vertical lines indicate *P* value and fold-change cut points, respectively. (**D**) Line graph illustrating the median expression of each identified spliceosome-related gene enriched in *AR*-nonmutated mCRPC tissue biopsies, relative to the median expression level of all expressed genes (*n* = 18,854) within the SU2C dataset (represented by the solid line). (**E**) Bubble plot showing the correlation between each identified spliceosome-related gene enriched in *AR*-nonmutated mCRPC tissue biopsies and both an AR and an AR-V7 mRNA signature score across the RMH transcriptomic cohort and the SU2C and WCDT datasets. The larger and darker the dot, the stronger the correlation between the respective gene and each mRNA signature score. (**F**) Heatmaps of gene expression show the level of TRA2B in mCRPC patient biopsies with and without detectable *AR* mutations within the RMH transcriptomic cohort, correlated with AR-V7 and AR signatures (17, 18). Samples ordered based on the AR-V7 signature, with expression values represented in transcripts per million (TPM). (**G** and **H**) Scatter plots displaying Pearson’s correlation (R) between AR-V7 mRNA level and the expression of TRA2B in mCRPC tissue biopsies without (**G**) and with (**H**) detectable *AR* mutations. RMH, Royal Marsden Hospital transcriptomic cohort; SU2C, International Stand Up To Cancer East Coast Dream Team cohort; WCDT, West Coast Dream Team cohort.

Several proteins involved in chromatin modification (epigenetic regulators) and spliceosome modulation (splicing factors) are implicated in generating AR-V7 (13–16). To investigate whether *AR*-mutant mCRPC exhibits reduced AR-V7 levels due to decreased alternative splicing activity, RNA-seq data from previously collected mCRPC biopsies with defined *AR* mutation status (RMH transcriptomic cohort) and from publicly available datasets (International Stand Up To Cancer East Coast Dream Team (SU2C) and West Coast Dream Team (WCDT) cohorts) were interrogated, and differences in global splicing events and splicing factor expression were determined. Overall, transcriptome data from 372 mCRPC patient tissue samples were analyzed from 3 independent cohorts: RMH transcriptomic cohort (*n* = 88), SU2C (*n* = 137), and WCDT (*n* = 147). Of these, 82 patients had detectable *AR* mutations (22%). Notably, evaluation of AR-V7 mRNA levels in *AR*-mutated and nonmutated mCRPC tissue biopsies from the SU2C dataset revealed *AR*-mutated cancers to exhibit lower levels of AR-V7 mRNA, corroborating the findings observed in the RMH clinical cohort (Supplemental Figure 3). Overall, no significant difference was observed in the total number of local splicing variations (LSVs) between tumors with or without *AR* mutation, suggesting that differences in AR-V7 levels between these 2 groups is not due to broadly altered global splicing (Supplemental Figure 4). Differences were, however, observed in the expression level of several spliceosome-related genes (Figure 4, A–C, and Supplemental Tables 4 and 5). To identify potential regulators of AR splicing among these, we first excluded genes with median expression levels in the lowest quartile of all expressed genes (based on the median expression level of all expressed genes within the SU2C dataset; Figure 4D). We then correlated the RNA expression of the most differentially overexpressed spliceosome-related genes in *AR*-nonmutated mCRPC biopsies, which have increased AR-V7, with previously validated AR and AR-V7 mRNA signature scores (14, 17). This revealed the serine and arginine rich (SR) splicing factor transformer-2 protein homolog beta (TRA2B), which is enriched in AR-nonmutated mCRPC biopsies, to strongly correlate with AR and AR-V7 activity across all 3 independent cohorts (Figure 4E), implicating TRA2B in *AR* pre-mRNA splicing. To test this hypothesis, RNA-Seq data from the RMH transcriptomic cohort was then further evaluated, revealing strong positive correlations between *TRA2B* expression and both AR-V7 (17) and AR (18) mRNA expression signatures in patients with and without detectable *AR* mutations (Figure 4F). Notably, however, while the expression of *TRA2B* correlated with AR-V7 mRNA levels in *AR*-nonmutated samples (*P* < 0.001), this was not the case in *AR*-mutated cases (*P* > 0.5; Figure 4, G and H). Taken together, these data reveal differences in splicing factor expression profiles between mCRPC biopsies with and without *AR* mutation and identify splicing factors that may be implicated in *AR* splicing and endocrine treatment-resistance, which merit further evaluation.

## Discussion

This study sought to elucidate why mCRPC with detectable *AR* mutations responds better to novel ARPIs in clinical development than mCRPC without detectable *AR* mutations. Herein, we demonstrate that mCRPCs with detectable *AR* mutations exhibit significantly lower levels of AR-V7, an alternatively spliced isoform of the AR associated with resistance to AR targeting therapies and worse prognosis. Interestingly, although specific *AR* mutations can confer resistance to ARPI therapies (10, 19), in the present study, mCRPC tissue biopsies with mutations of the *AR* LBD associated with better OS and improved response to ARPIs. While these findings are based on analyses of retrospective data from a limited patient cohort, which is a limitation of this study, our data support routine evaluation for these deleterious alterations (10, 19, 20).

*AR* mutations can be detected relatively easily by NGS of cell-free DNA (cfDNA), which offers ease of access and improved capture of genomic heterogeneity compared with mCRPC biopsy NGS studies. However, cfDNA mutation detection can associate with higher disease burden (21, 22) and potentially skew the results of clinical correlations, making the methods adopted in the present study better suited to elucidating clinical outcome associations. Furthermore, many studies of cfDNA group *AR* gene alterations, including mutations and amplifications (10, 23). While this improves sample size and power it renders the clinical significance of mutations difficult to interpret, since ctDNA *AR* amplification is associated with poorer prognosis and treatment resistance (24–26). In the present study, *AR* amplification was detected in both *AR*-mutated and nonmutated mCRPC tissue biopsies, indicating that these are not mutually exclusive genomic alterations. However, given the observation that *AR*-nonmutated mCRPC biopsies exhibit a higher incidence of *AR* amplification, this cannot be completely excluded as a contributing factor to the differences in clinical outcome observed between these 2 groups. Taken together, these findings support the evaluation in clinical trials of *AR* mutation status as a predictive biomarker of response to ARPIs targeting AR signaling driven by ligand binding, including both established agents (e.g., darolutamide after abiraterone) and emerging therapeutics such as AR degraders and CYP11A inhibitors.

*AR* gene copy number has been previously reported to drive AR-V7 production, with AR-V7 expression being promoted by *AR* gene amplification (12). In keeping with these reports, the studies herein indicate that AR-V7 levels are significantly higher in *AR-*nonmutated mCRPC biopsies with *AR* amplification than those without *AR* amplification. Interestingly, however, this was not the case in the mCRPC biopsies with detectable *AR* mutations evaluated in this study. Furthermore, we observed no difference in the total number of alternative splicing events in *AR*-mutated mCRPC biopsies compared with those without detectable *AR* mutation, suggesting that these observed differences in AR-V7 levels are not due to broadly altered global splicing. Taken together, these findings allude to the presence of other factors regulating AR-V7 generation. Several splicing and epigenetic regulators have been previously reported to be important for AR-V7 generation, including RBMX (13), JMJD6 (14), and SF3B1 (15). Consistent with these reports, we observed notable differences in splicing factor expression profiles between mCRPC biopsies with and without detectable *AR* mutations. In particular, we have found the splicing factor TRA2B to be overexpressed in mCRPC tissue biopsies without detectable AR mutations, with TRA2B RNA expression strongly correlating with AR-V7 activity. In keeping with these findings, TRA2B has recently been reported to regulate the splicing of *AR* transcripts in vitro, facilitating the synthesis of AR splice variants at the expense of full-length AR isoforms (27). Better understanding of the mechanisms regulating AR splicing is now needed to develop novel therapeutic strategies that can overcome oncogenic AR-V7 signaling and improve clinical outcomes from lethal prostate cancer.

### Conclusion.

*AR*-mutated mCRPC is associated with better clinical outcomes and greater ARPI sensitivity, at least, in part, due to significantly lower AR-V7 expression in these cancers. This appears to be independent of differences in global splicing but may be related to differences in splicing factor expression profiles between *AR*-mutated and nonmutated PCs.

## Methods

### Sex as a biological variant

This study included clinical biopsies from only male patients with a diagnosis of PC, given that PC arises in the prostate gland, which is a male-specific organ. The findings are therefore specific to PC in males.

### Patients

*RMH* clinical cohort. Interrogation of previously generated targeted Next Generation Sequencing (NGS) data from 475 mCRPC patient biopsies identified 68 patients with AR mutations, of which 58 had sufficient previously collected FFPE mCRPC tissue available for assessment (Bone, *n* = 17; Lymph node, *n* = 32; Liver, *n* = 4; Other, *n* = 5). Of the remaining 407 patients without detectable *AR* mutations, 64 had sufficient previously collected tissue and clinical data available for comparison (Bone, *n* = 32; Lymph node, *n* = 24; Liver, *n* = 4; Prostate, *n* = 4). Total RMH clinical cohort = 122 patients. All biopsy blocks were freshly sectioned and only considered for IHC analyses if adequate material was present (≥ 50 tumor cells). All diagnostic biopsies demonstrated adenocarcinoma. Patient clinical data were retrospectively collected from the RMH electronic patient record system.

#### RMH transcriptomic cohort.

Previously described patient transcriptome data from 95 fresh mCRPC tissue biopsies were analyzed (EGA accession number: EGAS50000001269) (28). Transcriptomes were aligned to the human reference genome (GRCh37/hg19) using TopHat2 (v2.0.7).

#### International Stand Up To Cancer East Coast Dream Team and West Coast Dream Team cohorts.

Previously described whole exome and transcriptome mCRPC patient sequencing data downloaded and reanalyzed (29, 30).

### Next generation sequencing

#### Sample acquisition and processing.

All PC biopsy samples were centrally reviewed by a pathologist. DNA was extracted from FFPE tumor blocks (average, 6 sections of 10 μm each per sample) using the FFPE Tissue DNA kit (Qiagen). DNA was quantified with the Quant-iT high-sensitivity PicoGreen double-stranded DNA Assay Kit (Invitrogen). The Illumina FFPE QC kit (WG-321-1001) was used for DNA quality control tests according to the manufacturer’s protocol as previously described (31).

#### Sequencing and bioinformatic analyses.

Libraries for next-generation targeted sequencing were constructed using a customized panel (GeneRead DNAseq Mix-n-Match Panel v2; Qiagen) covering 6,025 amplicons (398,702 bp) across 113 genes as per previously published methods (32, 33). Libraries were run using the MiSeq Sequencer (Illumina). FASTQ files were generated using the Illumina MiSeq Reporter v2.5.1.3. Sequence alignment and mutation calling were performed using the Qiagen GeneRead Targeted Exon Enrichment Panel Data Analysis Portal (https://ngsdataanalysis.qiagen.com). Mutation calls were reviewed manually in Integrative Genomics Viewer (https://software.broadinstitute.org/software/igv) according to the standard operating procedure for somatic variant refinement of tumor sequencing data, following principles previously described (34). Copy number variations (CNVs) in prostatic biopsies were assessed using the CNVkit (v0.3.5, https://github.com/etal/cnvkit) (35), which we have previously validated in an independent cohort of prostate cancer samples (36).

### Antibody validation

Antibody specificity was determined by Western blot (WB) analyses on whole cell lysates cultured with either nontargeting control siRNA or ON-TARGETplus pooled siRNA (Dharmacon; GE healthcare) as previously described (17). Antibodies listed in Supplemental Table 6.

### Immunohistochemistry

#### AR NTD.

IHC for AR NTD was performed as per previously described methods (37), or antigen retrieval was conducted for 10 minutes using Bond ER2 solution (Leica Biosystems), followed by incubation of the tissue with anti-AR antibody (diluted 1:1,000) for 15 minutes, before visualization of the reaction using the Bond Polymer Refine system (Leica Biosystems); VCaP and PC3 cells were used as controls. Mouse IgGs were used as negative controls.

#### AR-V7.

IHC for AR-V7 was performed as per previously described methods (17), or antigen retrieval was conducted for 10 minutes using Bond ER2 solution (Leica Biosystems), followed by incubation of the tissue with anti-AR-V7 antibody (diluted 1:500) for 30 minutes. Visualization of the reaction was achieved using the Bond Polymer Refine system (Leica Biosystems). 22RV1, VCaP, and PC3 cells were used as controls; Rabbit IgGs were used as negative controls. AR and AR-V7 quantification was determined by a pathologist blinded to clinical data using the H-score method (38); [(% of weak staining) × 1] + [(% of moderate staining) × 2] + [(% of strong staining) × 3], to determine overall percentage positivity across the stained tumor sample (range: 0 to 300).

### Whole transcriptome local splicing variation analysis

Whole transcriptome alternative splicing was investigated using MAJIQ (v2.5.6) as previously described (39). Local splicing variations (LSVs) were quantitated, and the splicing event type and PSI (percent spliced in) value determined. The total number of events of each type was then reported and the difference between groups was assessed using the Wilcoxon test for independent unpaired samples. Additionally, the heterogen module was used with default parameters to detect LSV changes between patients with *AR* mutation and without.

### Spliceosome related gene set

The spliceosome related gene set was curated from previous research (14) and supplemented with genes identified through searches in the Gene Ontology (GO), Kyoto Encyclopedia of Genes and Genomes (KEGG), and REACTOME databases (40–42). This resulted in a total of 428 genes, listed in Supplemental Table 4.

### Meta-analyses of differentially expressed genes

Differential gene expression analysis (DE) was conducted using RNA-Seq raw feature counts with DESeq2 (43) version 1.44.0. Bootstrapping was utilized to analyze differential gene expression. 500 iterations of resampling with replacement from the original dataset were performed. DE analysis was independently conducted on each resampled dataset. The results were aggregated by computing the median across all iterations. Meta-analyses of the DE results from the RMH transcriptomic, SU2C, and WCDT cohorts were conducted using Fisher’s method, the inverse normal method, and the fused inverse normal method. The fused inverse normal method was applied as previously described (44). Fisher and Inverse Normal were conducted using metaRNAseq (45) package version1.0.7, and results were corrected using Benjamin Hochberg method.

### AR-V7 and AR signature score

AR-V7 and AR signatures were generated using previously published methods (17, 18, 46, 47). For correlations with AR-V7 mRNA expression, AR-V7 levels were quantified by AR-V7 spliced reads per million (SRPM), as per previously published methods (26).

### Statistics

All statistical analyses were performed using Stata (v13.1), GraphPad Prism (v9.3.1 or v10.4.1), or R (v4.4.0), via the RStudio development environment (v2022.07.0, RStudio Team, 2022) and are listed in the relevant figure or table legend. H-Scores are reported as median values and interquartile ranges. Comparisons between *AR* mutation status and AR or AR-V7 expression levels in mCRPC tissue samples were made using Mann-Whitney test. Comparisons between 3 or more groups were performed using Kruskal-Wallis testing followed by Dunn’s correction for multiple comparisons. Comparison between *AR* mutation status and *AR* copy number in mCRPC tissue samples were made using Mann-Whitney test. Survival analyses were estimated using the Kaplan-Meier method, with hazard ratio determined by log-rank method. Differences between groups for bioinformatic analyses were evaluated using the Kruskal-Wallis test unless otherwise specified.

### Study approval

All patients had mCRPC treated at the RMH and provided written informed consent, being enrolled into protocols approved by the RMH ethics review committee (reference no. 04/Q0801/60).

### Data availability

All data values are included in the Supporting Data Values file. Transcriptomic and targeted sequencing datasets used in this study have been previously published and made available (29, 30, 36). The RMH transcriptomic cohort data are available via EGA accession number: EGAS50000001269 (28). The SU2C cohort data are available in cBioPortal and on GitHub under the code prad_su2c_2019. The WCDT cohort data are available on dbGaP with study number phs001648. The code used in this manuscript is available at: https://github.com/arianlundberg/AR_splicing

## Author contributions

AP, AS, and JdB were involved in research conceptualisation, data interpretation, and manuscript preparation. AP, IF, DB, AL, RS, BG, TT, OL, AF, CB, AN, JW, WY, CM, SRP, MCH, AGS, SC, and LG were involved in data collection, interpretation, and manuscript review. CM, MCH, and AS were involved with data validation, interpretation and manuscript review. NB, RN, LW, LG and SRP were involved in manuscript review.

## Funding support

- Prostate Cancer UK.
- The Movember Foundation through the London Movember Centre of Excellence (CEO13_2-002).
- The John Black Charitable Foundation.
- Prostate Cancer Foundation (18CHAL06 and 20YOUN17 [AP]).
- Cancer Research UK (Centre Programme grant).
- Experimental Cancer Medicine Centre grant funding from Cancer Research UK and the Department of Health.
- Cancer Research UK Convergence Science Centre (CTRQQR-2021\100009).
- Biomedical Research Centre funding to the Royal Marsden.
- The Wellcome Trust (AS).
- 2I01BX003324 Veterans Affairs R and D (SP).
- Cancer Research UK Newcastle Centre (C9380/A25138) (NB).
- Prostate Cancer Research (PCR-6955) (RN and LW).
- JdB is a National Institute for Health Research (NIHR) Senior Investigator.

## Supplementary Material

Supplemental data

Supporting data values

## Version 1 04/01/2026

Electronic publication
