# Supplementary material for: Androgen receptor splice variant 7 expression levels distinguish AR-mutated from nonmutated metastatic castration-resistant prostate cancers
Source: J Clin Invest. 2026 Apr 1;136(7):e198193. doi: 10.1172/JCI198193 (PMC13038193; doi:10.1172/JCI198193)
Supplement: Supplemental data [file jci-136-198193-s321.pdf]

Supplementary Figure 1: RMH cohort

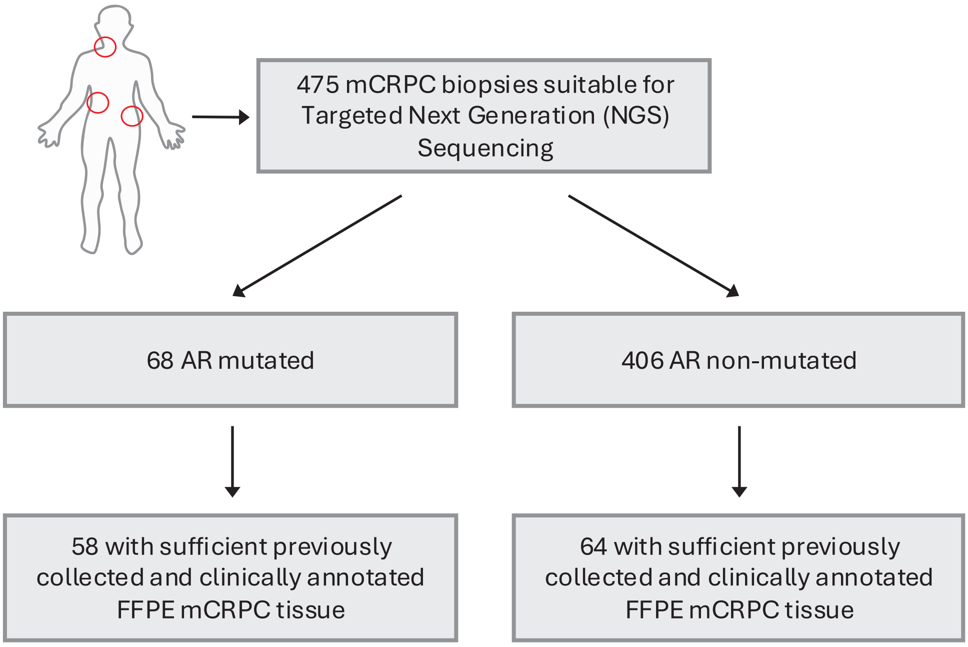

Supplementary Figure 1: RMH cohort consort diagram.

Supplementary Figure 2: Impact of AR-V7 status on clinical outcome in patients with and without detectable AR LBD mutations

A

Time on first ARPI

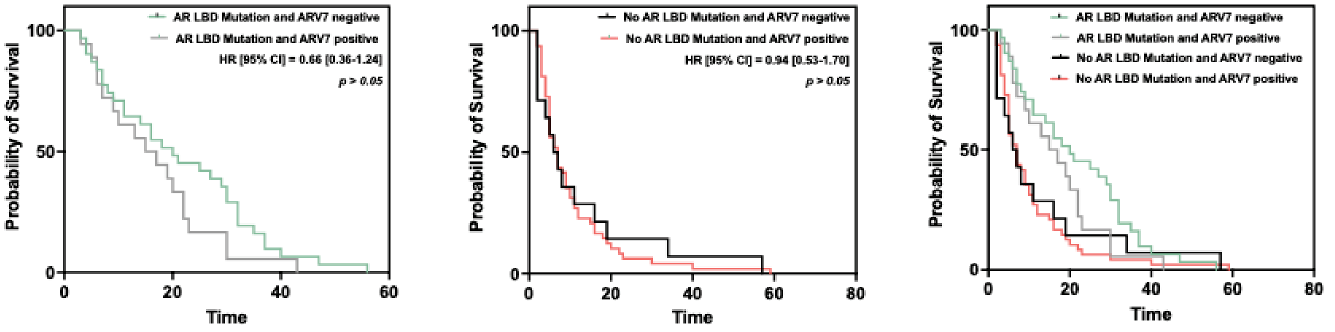

B

Overall survival from diagnosis

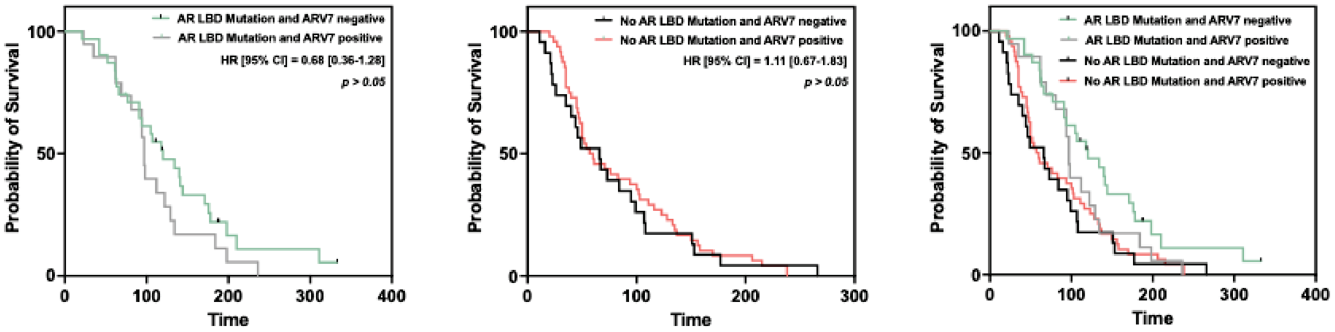

C

Overall survival from development of CRPC

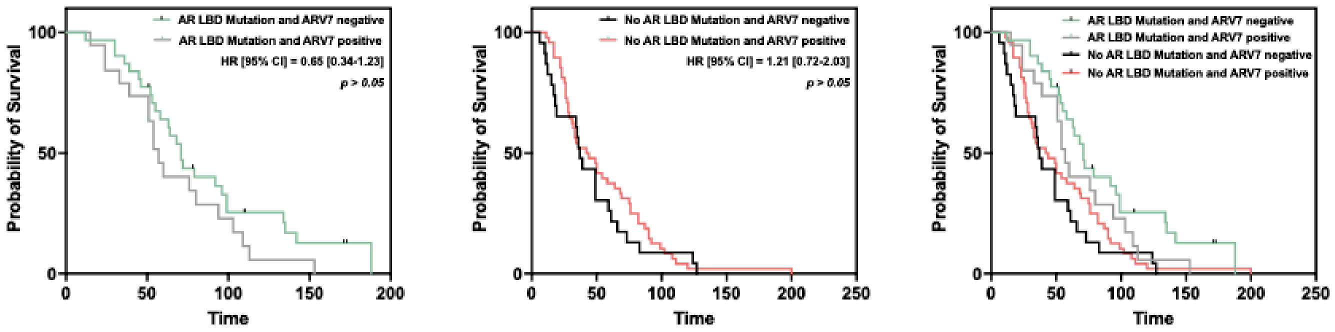

Supplemental Figure 2: Impact of AR-V7 status on clinical outcome in patients with and without detectable AR LBD mutations. (A-C) Kaplan-Meier curves comparing time on first ARPI (A), Overall survival from diagnosis (B), and overall survival from development of CRPC (C) between patients with and without detectable *AR* LBD mutation *(RMH clinical cohort)*.

Supplementary Figure 3: AR-V7 mRNA expression in AR mutated and non-mutated mCRPC biopsies (SU2C cohort)

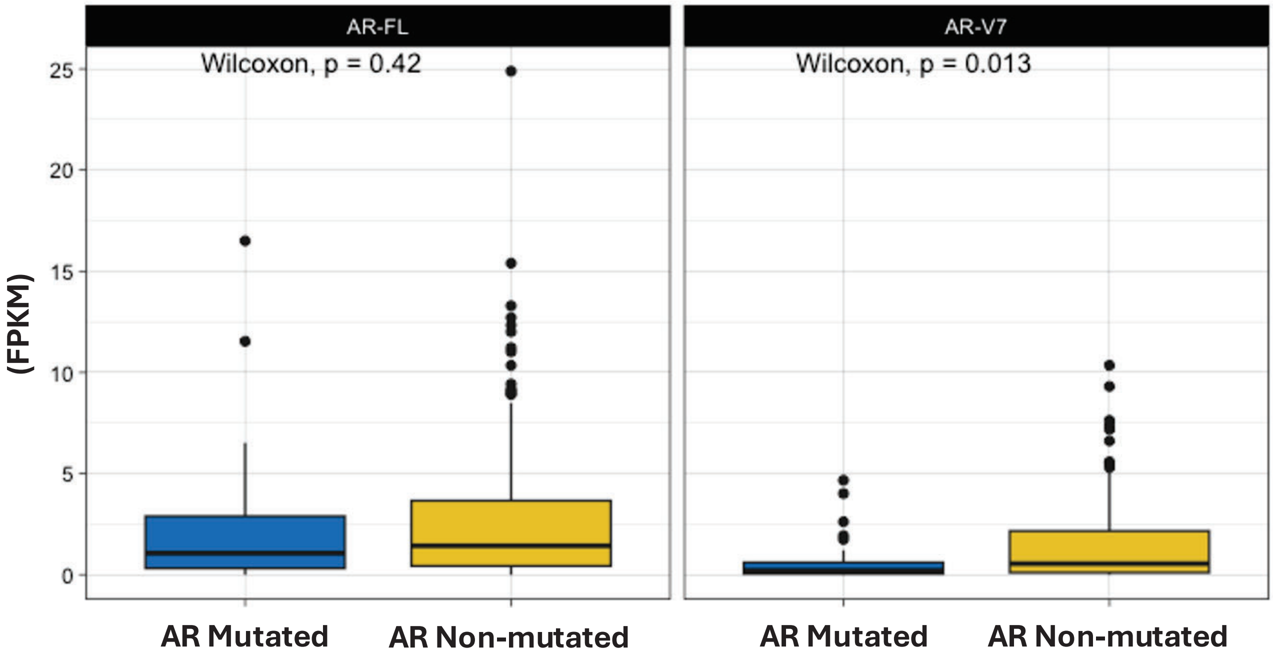

Supplemental Figure 3: AR-V7 mRNA expression in AR mutated and non-mutated mCRPC biopsies (SU2C cohort). (A-B) Box plots showing the expression level of AR and AR-V7 in mCRPC clinical biopsies with and without AR mutations in the publicly available SU2C dataset. *AR-FL = Androgen Receptor-Full length; AR-V7 = Androgen Receptor-Variant 7*.

Supplementary Figure 4: Meta-analysis of global splicing events in AR mutated verses non-mutated prostate cancer

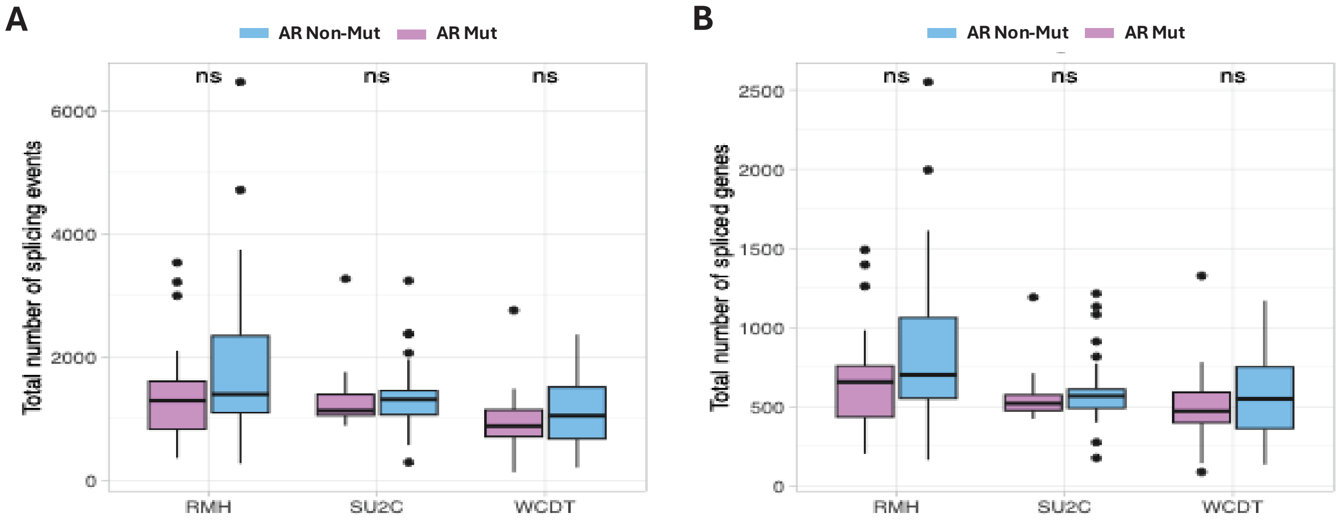

Supplemental Figure 4: Global splicing differences between mCRPC tumors with and without detectable AR mutation in three independent cohorts. (A-B) Box plots showing the total number of local splicing variations (LSVs) identified in for each patient (A) and the total number of genes the identified LSVs belong to (B) in patients with (purple) and without (blue) detectable AR mutation, across three independent mCRPC patient cohorts; RMH, SU2C and WCDT.

Supplementary Table 1: AR Mutation Type

AR Protein Annotation Change	No. Patients with Mutation	OncoKb Annotation	No. in Cosmic	ClinVar Somatic Annotation	Mutation Type
**T878A**	20	Oncogenic	104	Pathogenic	missense_variant
**F877L**	4	Likely Oncogenic	10	Uncertain significance	missense_variant
**L702H**	19	Likely Oncogenic	86	Likely Pathogenic	missense_variant
**H875Y**	13	Oncogenic	110	Pathogenic	missense_variant
**T878S**	2	Likely Oncogenic	6	Pathogenic	missense_variant
**A159T**	1	Inconclusive	2	Conflicting classifications of pathogenicity; Likely pathogenic(1); Benign(1)	missense_variant
**Q876L**	1	Unknown	0	-	missense_variant
**P380R**	1	Inconclusive	0	Conflicting classifications of pathogenicity; Uncertain significance(2); Benign(1); Likely benign(1)	missense_variant
**E355Q**	1	Likely Oncogenic	0	Conflicting classifications of pathogenicity ; Pathogenic(1); Benign(1)	missense_variant
**A646D**	2	Likely Oncogenic	7	Conflicting classifications of pathogenicity ; Uncertain significance(1); Benign(2); Likely benign(2)	missense_variant
**H875_L881del**	1	Likely Oncogenic	0	-	inframe_deletion
**V132I**	1	Likely Oncogenic	2	-	missense_variant
**V867A**	1	Likely Oncogenic	0	-	missense_variant
**G216R**	1	Likely Neutral	0	Likely Benign	missense_variant
**D891H**	1	Unknown	2	-	missense_variant
**M750T**	1	Unknown	2	-	missense_variant
**S889G**	1	Unknown	5	-	missense_variant
**V716M**	1	Unknown	8	-	missense_variant
**c.-16T>G**	1	Unknown	0	-	Start_gain

Clinical Characteristics		
	**AR Mutated**	**AR Non-mutated**
**Median age at diagnosis (years)**	62	63
**Gleason score (N, %)**		
<7	3, 5%	1, 2%
7	10, 17%	10, 16%
>7	34, 59%	41, 64%
NR	11, 19%	12, 19%
**Metastatic at diagnosis (N, %)**		
M0	6, 10%	5, 8%
M1	47, 81%	29, 45%
NR	5, 9%	30, 47%
**Initial treatment intent (N, %)**		
Radical	18, 31%	18, 28%
Palliative	38, 66%	36, 56%
Unknown	2, 3%	10, 16%
**Biopsy site (N, %)**		
Bone	17, 29%	32, 50%
Lymph node	33, 57%	24, 38%
Other	8, 14%	8, 12%
**Previous ARSI therapy**		
Abiraterone	26, 45%	35, 55%
Enzalutamide	26, 45%	22, 34%
Both	6, 10%	3, 5%
No previous ARSI	0, 0%	4, 6%

Supplementary Table 2: Table of patient characteristics.

Supplementary Table 3: Breakdown of AR amplification by mCRPC tissue biopsy site

	Biopsy site (Number of biopsies)				
	Bone	Lymph Node	Liver	Other	Total
AR mutated	17	32	4	5	58
AR non-mutated	32	24	4	4	64

	Biopsy site (% of biopsy samples)			
	Bone	Lymph Node	Liver	Other
AR mutated	29.3%	55.2%	6.9%	8.6%
AR non-mutated	50.0%	37.5%	6.3%	6.3%

	Number of samples with AR amplification				
	Biopsy site				
	Bone	Lymph Node	Liver	Other	Total
AR mutated	17	32	4	5	58
AR non-mutated	32	24	4	4	64

	% of biopsies			
	Biopsy site			
	Bone	Lymph Node	Liver	Other
AR mutated	29.3%	55.2%	6.9%	8.6%
AR non-mutated	50.0%	37.5%	6.3%	6.3%

**Supplementary Table 3: Spliceosome Related Genes Set**									
AAR2	CWC15	GCFC2	KHDC4	NSRP1	PRMT7	RBM8A	RSRC1	SNRPE	TIA1
ACIN1	CWC22	GEMIN2	KHDRBS1	NUDT21	PRPF18	RBMX	SAP18	SNRPF	TRA2A
ADAR	CWC25	GEMIN4	KHDRBS2	NUP98	PRPF19	RBMX2	SART1	SNRPG	TRA2B
AKAP17A	CWC27	GEMIN5	KHDRBS3	OVAAL	PRPF3	RBMXL1	SART3	SNRPGP15	TTF2
ALYREF	CWF19L1	GEMIN6	KHSRP	PABPC1	PRPF31	RBMXL2	SCAF11	SNRPN	TXNL4A
API5	CWF19L2	GEMIN7	LARP7	PABPN1	PRPF38A	RBMXL3	SCNM1	SNU13	TXNL4B
AQR	DAZAP1	GEMIN8	LGALS3	PAPOLA	PRPF38B	RBMY1A1	SETX	SNUPN	U2AF1
BCAS2	DBR1	GPATCH1	LINC01715	PCBP1	PRPF39	RBMY1B	SF1	SNW1	U2AF1L4
BUD13	DCPS	GPKOW	LMNTD2	PCBP2	PRPF4	RBMY1F	SF3A1	SON	U2AF2
BUD31	DDX1	GTF2F1	LOC100996657	PCBP4	PRPF40A	RBMY1J	SF3A2	SPEN	U2SURP
C1QBP	DDX17	GTF2F2	LSM1	PCF11	PRPF40B	REST	SF3A3	SREK1	UBL5
C7orf55-LUC7L2	DDX20	HELB	LSM2	PDCD7	PRPF4B	RHEB	SF3B1	SRPK1	UHMK1
C9orf78	DDX23	HMX2	LSM3	PHF5A	PRPF6	RNF113A	SF3B2	SRPK2	UPF1
CACTIN	DDX39A	HNRNPA0	LSM4	PLRG1	PRPF8	RNF113B	SF3B3	SRPK3	UPF3B
CASC3	DDX39B	HNRNPA1	LSM5	PNN	PSIP1	RNPC3	SF3B4	SRRM1	USP39
CCAR1	DDX41	HNRNPA1L2	LSM6	POLR2A	PSPC1	RNPS1	SF3B5	SRRM2	USP4
CCDC12	DDX42	HNRNPA1P6	LSM7	POLR2B	PTBP1	RNU1-4	SF3B6	SRRM4	USP49
CD2BP2	DDX46	HNRNPA2B1	LSM8	POLR2C	PTBP2	RNU11	SFPQ	SRRT	WAC
CDC40	DDX5	HNRNPA3	LUC7L	POLR2D	PUF60	RNU2-1	SFSWAP	SRSF1	WBP11
CDC5L	DGCR14	HNRNPC	LUC7L2	POLR2E	QKI	RNU4-1	SKIV2L2	SRSF10	WBP4
CDK13	DHX15	HNRNPD	LUC7L3	POLR2F	RALY	RNU4-2	SLC39A5	SRSF11	WDR33
CELF1	DHX16	HNRNPF	MAGOH	POLR2G	RAVER1	RNU4ATAC	SLU7	SRSF12	WDR77
CELF2	DHX32	HNRNPH1	MAGOHB	POLR2H	RAVER2	RNU5A-1	SMC1A	SRSF2	WDR83
CELF3	DHX35	HNRNPH2	MBNL1	POLR2I	RBFOX1	RNU5B-1	SMN1	SRSF3	WEE2-AS1
CELF4	DHX38	HNRNPH3	MBNL2	POLR2J	RBFOX2	RNU5D-1	SMN2	SRSF4	WTAP
CELF5	DHX8	HNRNPK	MBNL3	POLR2K	RBFOX3	RNU5E-1	SMNDC1	SRSF5	XAB2
CELF6	DHX9	HNRNPL	METTL14	POLR2L	RBM10	RNU5F-1	SMU1	SRSF6	YBX1
CHERP	DNAJC8	HNRNPM	METTL16	PPAN	RBM11	RNU6-1	SNIP1	SRSF7	YJU2
CIRBP	DQX1	HNRNPR	METTL3	PPARGC1A	RBM15	RNU6-7	SNRNP200	SRSF8	YJU2B
CLNS1A	DYRK1A	HNRNPU	MFAP1	PPIE	RBM15B	RNU6-9	SNRNP25	SRSF9	YTHDC1
CLP1	EFTUD2	HNRNPUL1	MTREX	PPIG	RBM17	RNU6ATAC	SNRNP27	STH	ZBTB7A
COIL	EIF4A3	HSPA1A	MYEF2	PPIH	RBM22	RNVU1-1	SNRNP35	STRAP	ZC3H10
CPSF1	ELAVL1	HSPA1B	MYOD1	PPIL1	RBM23	RNVU1-14	SNRNP40	SUGP1	ZCCHC8
CPSF2	ELAVL2	HSPA1L	NAA38	PPIL3	RBM24	RNVU1-15	SNRNP48	SYF2	ZCRB1
CPSF3	ESRP2	HSPA2	NCBP1	PPIL4	RBM25	RNVU1-17	SNRNP70	SYMPK	ZMAT2
CPSF4	ESS2	HSPA6	NCBP2	PPIL6	RBM28	RNVU1-19	SNRPA	SYNCRIP	ZMAT5
CPSF7	FAM172A	HSPA8	NCBP2L	PPP1R8	RBM3	RNVU1-2A	SNRPA1	TCERG1	ZNF638
CRNKL1	FIP1L1	HTATSF1	NCL	PPWD1	RBM39	RNVU1-3	SNRPB	TFIP11	ZNF830
CSTF1	FMR1	IK	NFX1	PQBP1	RBM4	RNVU1-4	SNRPB2	TGS1	ZRANB2
CSTF2	FRG1	ISY1	NOL3	PRCC	RBM41	RNVU1-6	SNRPC	THOC1	ZRSR2
CSTF2T	FUS	IVNS1ABP	NONO	PRDX6	RBM42	RNVU1-7	SNRPD1	THOC2	ZRSR2P1
CSTF3	FXR1	JMJD6	NOVA1	PRKRIP1	RBM5	RNVU1-8	SNRPD2	THOC3	
CTNNBL1	FXR2	KDM1A	NOVA2	PRMT5	RBM7	RP9	SNRPD3	THRAP3	

Supplementary Table 4: Meta-analysis Differential Eexpression

Genes	logFC	metaP	metaFDR	log10p
RNVU1-8	1.53064683	8.70792E-06	0.001230719	5.060085736
GEMIN6	0.100000298	0.002689047	0.099364591	2.570401629
RNU5F-1	0.072964156	0.02936342	0.289139871	1.532193365
USP49	0.03992839	0.000117573	0.008308488	3.929692601
RBMXL2	0.06332595	0.044993123	0.308124355	1.346853864
FMR1	0.0974481	0.012249753	0.172845286	1.911872661
MBNL1	0.074791812	0.032918977	0.289139871	1.48255367
RHEB	0.075620712	0.034364246	0.289139871	1.463893179
PAPOLA	0.082348107	0.022895493	0.255465503	1.640249997
SNRPE	0.094385176	0.014480922	0.180585613	1.839203793
SNRNP27	0.063688768	0.06066108	0.352332848	1.217089862
WBP4	-0.002217565	0.006946563	0.140254409	2.158230039
RNU6-1	0.028715833	0.054327395	0.333837904	1.264981121
RNU2-1	0.010922059	0.014059035	0.180585613	1.852044489
SNW1	0.061283703	0.052091887	0.332601933	1.283229907
TRA2B	0.10450484	0.004102573	0.11127421	2.386943708
RBMY1A1	0.037202953	0.221500392	0.518516996	0.654625501
SLU7	0.059843457	0.115979082	0.423923543	0.935620331
MAGOHB	0.038566763	0.041385196	0.30784777	1.383154988
POLR2K	0.081373177	0.008299417	0.146623026	2.080952438
RBM25	0.078341895	0.032894678	0.289139871	1.482874362
RBM10	-0.008287276	0.007367621	0.141994159	2.132672695
QKI	0.059437228	0.079274296	0.395438843	1.100867604
LSM3	0.052929926	0.060125071	0.352332848	1.220944395
HNRNPU	0.006935048	0.067408351	0.376067644	1.171286295
HSPA2	0.083315869	0.009198837	0.150011805	2.036267071
GTF2F2	0.018210813	0.083956275	0.406816521	1.075946836
RBMY1F	0.029115123	0.360013943	0.57798646	0.443680679
DHX32	-0.042977892	0.124815766	0.444721722	0.903730552
RNU6-9	0.030412407	0.0626919	0.356122514	1.202788565
JMJD6	-0.09280693	0.011382868	0.166425387	1.943748282
RBMY1B	0.023621748	0.41520282	0.586406907	0.381739705
SNRNP48	-0.005326721	0.084433618	0.406816521	1.073484602
DHX16	-0.017964165	0.109932278	0.42228249	0.958874771
SNRPB2	0.013604365	0.100306821	0.42228249	0.998669533
RBMY1J	0.025391166	0.416639953	0.586406907	0.380239086
METTL3	-0.038324672	0.184896343	0.511444571	0.733071679
U2AF2	-0.009318494	0.078208256	0.395438843	1.106747398
SF1	-0.01219269	0.157572552	0.476850978	0.802519432
SRRM4	0.542366585	3.14903E-05	0.002670376	4.501823383
PABPN1	0.010599746	0.053015168	0.332601933	1.275599858
CWC22	0.04257091	0.167621764	0.486791972	0.775669593
ESRP2	-0.061019392	0.071308404	0.383684539	1.146859284
PPWD1	0.019785611	0.210255582	0.518516996	0.677252465
MBNL2	0.128081091	0.003029203	0.099364591	2.518671566

**RBM15B**	-0.078789169	0.044003509	0.308124355	1.356512689
**DQX1**	-0.056337136	0.000419433	0.022229934	3.377337699
**SRSF7**	-0.006568454	0.039966387	0.307359725	1.398305111
**ZC3H10**	-0.039726221	0.201415753	0.518516996	0.695906566
**NOVA1**	0.041239565	0.042665406	0.308124355	1.369924112
**IK**	0.029422894	0.20219272	0.518516996	0.694234486
**GEMIN8**	-0.016010273	0.070110898	0.383684539	1.15421447
**KHDRBS2**	0.086661203	0.012637273	0.172845286	1.898346622
**SREK1**	0.01461264	0.259233944	0.540285545	0.586308133
**RNU1-4**	0.003835574	0.131424623	0.450114114	0.881323259
**RNVU1-7**	0.010110429	0.078971143	0.395438843	1.102531577
**CWF19L2**	-0.012235196	0.080503689	0.396901908	1.094184218
**DAZAP1**	-0.018755012	0.294034237	0.560381966	0.531602098
**PHF5A**	0.06781272	0.089639649	0.42228249	1.047499855
**CELF4**	0.48365394	8.66669E-06	0.001230719	5.062146588
**RNU5B-1**	0.047299885	0.154514241	0.471538755	0.811031488
**SRSF3**	0.036332529	0.043460988	0.308124355	1.361900404
**PRMT7**	-0.037831269	0.24632535	0.538148254	0.608490892
**ISY1**	-0.029566767	0.282585391	0.558902963	0.548850294
**ZRSR2**	-0.001821853	0.220204375	0.518516996	0.657174058
**U2SURP**	-0.016804486	0.05017129	0.332384797	1.299544731
**FXR1**	0.008718724	0.160676302	0.476850978	0.794048172
**RBM22**	0.031806036	0.341214121	0.577230597	0.466973004
**MBNL3**	0.033798738	0.193751288	0.518516996	0.712755403
**RNU11**	0.028348938	0.344393695	0.577230597	0.462944808
**SRSF12**	-0.04481137	0.222094612	0.518516996	0.653461978
**HTATSF1**	0.09172453	0.017016994	0.20042237	1.769117163
**RNPC3**	0.018970678	0.22048661	0.518516996	0.65661778
**UHMK1**	0.039588562	0.211987776	0.518516996	0.673689181
**RBMXL3**	0.022222894	0.110550369	0.42228249	0.956439804
**SLC39A5**	0.045378127	0.002446543	0.099364591	2.611447079
**API5**	0.085676373	0.007755138	0.142964282	2.110410474
**LMNTD2**	-0.043091448	0.113675742	0.423923543	0.944332201
**SYF2**	-0.01275497	0.124286083	0.444721722	0.9055775
**PCBP4**	-0.054980537	0.076736172	0.395438843	1.114999867
**POLR2D**	-0.02384199	0.406317919	0.586406907	0.391134024
**PRCC**	-0.035046874	0.248763653	0.538148254	0.604213075
**KDM1A**	-0.058964662	0.161949389	0.476850978	0.790620687
**HNRNPA3**	0.05355458	0.034548647	0.289139871	1.461568954
**PRPF40B**	-0.013041066	0.151402349	0.470031869	0.819867386
**PQBP1**	-0.039629855	0.196300039	0.518516996	0.707079614
**TFIP11**	0.056057793	0.107268809	0.42228249	0.969526543
**GEMIN4**	-0.036923502	0.331750067	0.577230597	0.47918898
**TXNL4A**	-0.020708851	0.36173466	0.57798646	0.441609877
**NFX1**	0.014278486	0.358153878	0.57798646	0.445930342
**RSRC1**	0.009510516	0.339502825	0.577230597	0.469156608
**THRAP3**	-0.098477206	0.003046556	0.099364591	2.516190855
**SNRPA1**	-0.008830882	0.242594224	0.538148254	0.615119544

**RNPS1**	-0.000629304	0.137984675	0.453531024	0.860169146
**RNU4-2**	0.01663825	0.105165763	0.42228249	0.978125622
**KHDC4**	-0.00946429	0.133853112	0.450114114	0.873371528
**RBMX2**	0.052948801	0.134731863	0.450114114	0.870529685
**WEE2-AS1**	-0.000121926	0.036824417	0.289139871	1.433864114
**ZNF830**	-0.022178334	0.299287378	0.560837059	0.523911598
**RP9**	-0.021337722	0.288678653	0.558902963	0.53958533
**FXR2**	-0.032365119	0.294096026	0.560381966	0.531510844
**DDX17**	0.018605786	0.341212135	0.577230597	0.466975532
**RNU6ATAC**	0.003254237	0.406049427	0.586406907	0.391421098
**FRG1**	0.059304929	0.129003874	0.450114114	0.889397247
**AKAP17A**	0.081035867	0.010692797	0.161919493	1.970908691
**RNU5D-1**	0.043988886	0.180184109	0.505947433	0.744283513
**HNRNPH2**	0.025787283	0.449414692	0.597341158	0.347352734
**OVAAL**	-0.014043831	0.466436879	0.601289043	0.331207119
**PSPC1**	-0.058768658	0.151873505	0.470031869	0.818517985
**TCERG1**	0.045246561	0.287427557	0.558902963	0.541471596
**C9orf78**	-0.020365013	0.400958865	0.586406907	0.39690018
**SNRNP70**	-0.027269912	0.312954279	0.574426903	0.504519105
**SNRPN**	-0.046049868	0.134821916	0.450114114	0.870239505
**PPARGC1A**	0.027030928	0.195241301	0.518516996	0.709428306
**THOC1**	-0.027710844	0.105438664	0.42228249	0.977000104
**RNU4-1**	0.042088258	0.090586745	0.42228249	1.042935344
**ZMAT2**	0.025122568	0.184296863	0.511444571	0.734482056
**CDC40**	0.007818101	0.346009792	0.577230597	0.460911611
**SRRM2**	-0.037743201	0.236620623	0.533655023	0.625947406
**CPSF2**	0.046960302	0.264642062	0.542068764	0.577341128
**NUDT21**	0.076928026	0.04505592	0.308124355	1.34624814
**HNRNPA2B1**	-0.018956412	0.109038927	0.42228249	0.962418429
**CWF19L1**	6.43256E-05	0.235573509	0.533655023	0.62787355
**SF3B4**	0.054191709	0.147818326	0.470031869	0.83027172
**SF3B1**	0.026766954	0.475701905	0.601289043	0.322665109
**DDX39B**	0.006049368	0.175670928	0.49771712	0.755300104
**RNU4ATAC**	0.021516621	0.145570288	0.467589411	0.836927258
**RBM8A**	-0.010594502	0.376645932	0.582021411	0.424066719
**TIA1**	-0.005198011	0.202073112	0.518516996	0.69449147
**CACTIN**	0.008304847	0.244539857	0.538148254	0.611650346
**UPF1**	-0.03651043	0.440085948	0.590921467	0.356462498
**RNU6-7**	0.000266326	0.418452441	0.586406907	0.378353895
**CSTF1**	0.066922819	0.112961378	0.423923543	0.947070019
**LUC7L**	-0.003089382	0.385433858	0.582495441	0.414050138
**AQR**	-0.019212157	0.092674477	0.42228249	1.033039854
**RNU5A-1**	0.018618342	0.384472509	0.582495441	0.415134708
**DHX9**	0.019927897	0.419059653	0.586406907	0.377724151
**BUD31**	0.010718422	0.40706918	0.586406907	0.390331777
**CELF1**	-0.007382172	0.172279728	0.496915679	0.763765822
**CPSF7**	-0.047518523	0.051874103	0.332601933	1.285049397
**SF3A2**	0.007462044	0.283958394	0.558902963	0.546745289

**FAM172A**	-0.010195927	0.254700159	0.539782018	0.593970783
**MYOD1**	0.03029539	0.098785758	0.42228249	1.005305665
**PRKRIP1**	-0.068715148	0.040594681	0.307359725	1.39153087
**NOL3**	-0.084051547	0.023665599	0.257287536	1.625882501
**USP39**	0.047224194	0.212145048	0.518516996	0.673367102
**LINC01715**	-0.030337209	0.255184799	0.539782018	0.593145199
**CELF3**	0.508558081	2.34443E-05	0.002485097	4.629962478
**RNF113B**	-0.008124763	0.212097248	0.518516996	0.673464967
**RBMX**	0.081628352	0.006723831	0.140254409	2.172383181
**SRSF10**	0.060064815	0.099965372	0.42228249	1.000150413
**BCAS2**	0.012421831	0.41803319	0.586406907	0.378789236
**SFPQ**	-0.026699274	0.435420374	0.590921467	0.361091253
**PNN**	-0.015594902	0.389309985	0.583277151	0.409704457
**WAC**	-0.017529442	0.222726127	0.518516996	0.652228834
**DDX42**	0.031007081	0.2571574	0.539782018	0.589800973
**HNRNPH1**	0.001266312	0.35274658	0.57798646	0.452537189
**REST**	-0.044568441	0.031754635	0.289139871	1.498192873
**CELF5**	-0.004636817	0.05334182	0.332601933	1.272932174
**RBM23**	0.031621394	0.465132862	0.601289043	0.332422976
**NOVA2**	0.420735949	7.26886E-06	0.001230719	5.138533866
**CD2BP2**	0.017513478	0.55972817	0.621003717	0.252022835
**ALYREF**	-0.015963116	0.45515205	0.601197723	0.341843497
**POLR2E**	-0.009072039	0.510593588	0.601289043	0.291924643
**PRPF3**	0.008451711	0.035565023	0.289139871	1.448976912
**SRRM1**	-0.031567911	0.269253599	0.543635838	0.569838483
**PRPF38B**	0.00216639	0.440403735	0.590921467	0.356149007
**LGALS3**	0.011333297	0.331538893	0.577230597	0.479465517
**SF3B6**	0.003197987	0.343532141	0.577230597	0.464032624
**SNRPG**	0.021075078	0.377490302	0.582021411	0.423094201
**PRMT5**	0.005537115	0.259948706	0.540285545	0.58511234
**SRSF8**	-0.073335825	0.05671427	0.343526434	1.246307656
**CDK13**	0.019427096	0.520290854	0.601289043	0.283753808
**WDR77**	-0.053469418	0.142420248	0.460963244	0.846428264
**CTNNBL1**	0.0036621	0.36669424	0.57798646	0.435695912
**RNVU1-1**	-0.049551104	0.115233352	0.423923543	0.938421804
**DDX46**	0.00691583	0.43081568	0.590921467	0.365708499
**THOC2**	0.004853315	0.186966765	0.511444571	0.728235585
**PPIG**	-0.019907229	0.151683539	0.470031869	0.819061548
**UBL5**	0.038182182	0.186802804	0.511444571	0.72861661
**MTREX**	0.027225062	0.445823159	0.596131237	0.350837375
**PTBP2**	-0.030703341	0.250163194	0.538307203	0.601776587
**DYRK1A**	0.038605731	0.326910121	0.577230597	0.485571633
**STRAP**	0.012693592	0.496062306	0.601289043	0.304463772
**BUD13**	-0.008055349	0.473852434	0.601289043	0.324356885
**KHDRBS3**	-0.02811912	0.071488393	0.383684539	1.145764466
**LUC7L3**	-0.036465316	0.232394268	0.532622538	0.633774589
**CLNS1A**	0.032454318	0.295450186	0.560381966	0.529515732
**ELAVL2**	0.0085747	0.408322186	0.586406907	0.388997022

**METTL16**	-0.005864486	0.515534099	0.601289043	0.287742604
**TGS1**	0.027716993	0.262311344	0.540465552	0.581182928
**HSPA6**	-0.06052169	0.030012175	0.289139871	1.522702535
**HNRNPA0**	-0.035120986	0.434435852	0.590921467	0.362074341
**RBFOX1**	0.048504339	0.008997692	0.150011805	2.045868892
**PPAN**	-0.021105656	0.584050903	0.62811387	0.2335493
**LSM5**	-0.000624279	0.408606373	0.586406907	0.388694864
**RBMXL1**	-0.059539511	0.127371948	0.450047548	0.89492621
**XAB2**	-0.018933332	0.486909321	0.601289043	0.312551912
**LSM2**	0.042911107	0.300259463	0.560837059	0.522503296
**PSIP1**	0.04147471	0.115427745	0.423923543	0.937689788
**SRSF6**	-0.005961538	0.362214217	0.57798646	0.441034507
**HSPA1L**	-0.04232027	0.101858503	0.42228249	0.99200271
**THOC3**	0.039914952	0.161557266	0.476850978	0.791673504
**PDCD7**	0.021636774	0.214584323	0.518516996	0.668402009
**FUS**	0.01943653	0.17607917	0.49771712	0.754292018
**SNRPGP15**	0.078138422	0.034423078	0.289139871	1.463150302
**LUC7L2**	-0.025200133	0.411549256	0.586406907	0.385578179
**PRDX6**	-0.008022639	0.268665288	0.543635838	0.570788441
**SMU1**	0.020367749	0.510421258	0.601289043	0.292071246
**IVNS1ABP**	0.0771878	0.020419899	0.234001	1.689946419
**CHERP**	0.000417834	0.497998595	0.601289043	0.302771882
**RNVU1-3**	-0.003286269	0.580583843	0.62811387	0.236135054
**HNRNPK**	0.000886405	0.5029277	0.601289043	0.298494444
**KHSRP**	-0.002693449	0.517799382	0.601289043	0.285838472
**HSPA1A**	-0.007031542	0.471763485	0.601289043	0.326275677
**LSM8**	0.001808902	0.200598056	0.518516996	0.697673279
**RNVU1-15**	0.029686277	0.173452466	0.496917876	0.760819521
**SNRPD1**	0.019222877	0.287515475	0.558902963	0.541338775
**POLR2A**	-0.028238077	0.324634168	0.577230597	0.488605772
**SRSF1**	-0.004410728	0.109942799	0.42228249	0.958833209
**NAA38**	0.018156867	0.2405223	0.536744502	0.618844651
**DBR1**	-0.011541703	0.347155194	0.577230597	0.459476333
**GPATCH1**	-0.013457697	0.645290171	0.662477077	0.19024495
**SNRPD2**	-0.005619419	0.363779557	0.57798646	0.43916171
**PRPF31**	-0.041288246	0.381737216	0.582495441	0.418235498
**SNRPD3**	-0.002430185	0.467914744	0.601289043	0.32983327
**SRSF9**	-0.043784524	0.299089818	0.560837059	0.524198371
**PPIL3**	0.017405946	0.588196337	0.629785977	0.230477684
**SPEN**	-0.047754969	0.219276907	0.518516996	0.659007104
**CCAR1**	-0.020302397	0.209308251	0.518516996	0.679213652
**NCBP2**	0.015802418	0.50433081	0.601289043	0.297284499
**RBM24**	-0.457837657	0.00017514	0.010608464	3.756615328
**CASC3**	-0.037716913	0.246800423	0.538148254	0.607654101
**NCBP2L**	-0.021731057	0.438126562	0.590921467	0.358400416
**HSPA1B**	-0.024819571	0.305318736	0.564174938	0.515246544
**PABPC1**	0.009106274	0.558718212	0.621003717	0.252807172
**RBM3**	-0.017279744	0.29605085	0.560381966	0.528633688

**SYMPK**	0.029684902	0.13774518	0.453531024	0.860923589
**TXNL4B**	0.049474683	0.074191596	0.388360949	1.129645284
**SAP18**	-0.036809625	0.107577224	0.42228249	0.968279667
**RBM7**	-0.017611911	0.334347615	0.577230597	0.475801771
**HMX2**	0.023005058	0.198522254	0.518516996	0.702190803
**CELF2**	0.199414771	0.003718735	0.11127421	2.429604821
**NCL**	0.006404988	0.536280621	0.614548603	0.270607897
**RBFOX3**	0.250541881	0.004199027	0.11127421	2.376851354
**PRPF40A**	-0.019221443	0.361186717	0.57798646	0.442268229
**SART1**	0.019467837	0.492568217	0.601289043	0.307533615
**SCNM1**	0.030469057	0.278643723	0.558902963	0.554950736
**CSTF2T**	0.039382818	0.306038292	0.564174938	0.514224231
**HNRNPR**	0.006007324	0.679850507	0.691262866	0.167586574
**LSM7**	0.006559791	0.408006687	0.586406907	0.389332719
**SF3B5**	-0.000887255	0.590872197	0.631057459	0.228506445
**PRPF6**	0.017286193	0.328613117	0.577230597	0.483315105
**YBX1**	0.022615353	0.354927956	0.57798646	0.449859792
**STH**	-0.00760961	0.457044062	0.601289043	0.340041929
**GEMIN7**	-0.01741316	0.327700425	0.577230597	0.484522996
**SNRNP35**	-0.028500511	0.382527971	0.582495441	0.417336803
**CLP1**	0.001319564	0.408320306	0.586406907	0.388999021
**MAGOH**	0.010611102	0.466726043	0.601289043	0.330937965
**RBM4**	0.025603131	0.131641107	0.450114114	0.880608476
**CRNKL1**	-0.036062982	0.333004114	0.577230597	0.477550401
**SF3A1**	0.023866311	0.208419363	0.518516996	0.681061935
**HNRNPC**	0.059379033	0.073692965	0.388360949	1.132573968
**LSM6**	-0.027432004	0.062993369	0.356122514	1.200705162
**HNRNPL**	-0.015643313	0.237964794	0.533846945	0.62348729
**POLR2H**	-0.000907184	0.544144482	0.620207689	0.26428577
**RNVU1-4**	0.008919244	0.602294347	0.638432008	0.220191213
**PPIH**	0.004207878	0.478823523	0.601289043	0.319824523
**SFSWAP**	0.042130088	0.10691053	0.42228249	0.970979516
**ADAR**	0.019391775	0.563713698	0.621003717	0.248941412
**CSTF2**	0.024028213	0.572106826	0.625188903	0.242522871
**ZCCHC8**	0.032992092	0.211157922	0.518516996	0.67539262
**CCDC12**	0.018387485	0.371789197	0.579316575	0.429703233
**PRPF18**	0.050953566	0.134613936	0.450114114	0.870909976
**PRPF39**	-0.00686011	0.515230112	0.601289043	0.287998763
**SNRNP200**	0.036895173	0.091620341	0.42228249	1.038008097
**RBM11**	0.019645368	0.229473938	0.528787771	0.639266631
**DCPS**	0.00027396	0.351668461	0.57798646	0.453866579
**POLR2L**	0.021667635	0.634981453	0.656663747	0.197238959
**ZNF638**	-0.024262544	0.235314719	0.533655023	0.628350907
**YTHDC1**	-0.00181236	0.560902887	0.621003717	0.251112324
**FIP1L1**	-0.051421131	0.034866254	0.289139871	1.457594705
**PUF60**	0.01416501	0.496565553	0.601289043	0.304023411
**RBM15**	0.017621422	0.503256713	0.601289043	0.298210423
**SRRT**	-0.004767855	0.583516058	0.62811387	0.233947188

**TTF2**	0.011758723	0.558113177	0.621003717	0.253277723
**DDX20**	0.002911435	0.248766646	0.538148254	0.60420785
**SNRPF**	0.03216838	0.218044425	0.518516996	0.661455012
**PCBP2**	-0.011161801	0.356304414	0.57798646	0.448178798
**RBM39**	0.00638401	0.550857268	0.621003717	0.258960916
**NONO**	0.02079695	0.058721578	0.350675338	1.231202284
**SNRPA**	0.013472254	0.336672724	0.577230597	0.472792067
**ZBTB7A**	-0.014867047	0.251379307	0.538307203	0.599670475
**GPKOW**	-0.001653617	0.517465527	0.601289043	0.286118577
**CDC5L**	-0.004111834	0.305366625	0.564174938	0.515178431
**HELB**	0.050884884	0.006328776	0.140254409	2.198680243
**NSRP1**	-0.043353264	0.118006324	0.427646848	0.92809472
**ACIN1**	-0.010727988	0.554826755	0.621003717	0.255842604
**EIF4A3**	-0.022910772	0.570869935	0.625188903	0.243462829
**SRSF2**	0.007491982	0.322887619	0.577230597	0.490948608
**RNF113A**	0.016556283	0.584746638	0.62811387	0.233032266
**PRPF4B**	-0.002147941	0.578242891	0.62811387	0.237889698
**POLR2F**	-0.009207907	0.497467564	0.601289043	0.303235231
**DDX39A**	0.047750402	0.099270697	0.42228249	1.003178928
**RBM41**	0.014452974	0.532118429	0.611431474	0.2739917
**WDR33**	-0.028034289	0.447098427	0.596131237	0.349596858
**GTF2F1**	-0.011342217	0.223793892	0.518516996	0.650151771
**SNRNP25**	-0.008905342	0.257160301	0.539782018	0.589796075
**GEMIN2**	0.026854006	0.320039615	0.577230597	0.494796261
**PRPF38A**	-0.019185025	0.539148015	0.61616916	0.26829199
**SMN1**	0.081180926	0.005789041	0.136364084	2.237393351
**PPIL6**	0.000721183	0.425242815	0.590921467	0.371363016
**SUGP1**	-0.024309663	0.461267272	0.601289043	0.336047358
**CPSF3**	0.004838467	0.593869562	0.632665061	0.226308933
**HNRNPM**	-0.01124753	0.667732849	0.680573866	0.175397258
**SNRNP40**	-0.03773842	0.369776128	0.579316575	0.43206113
**WDR83**	-0.002506812	0.404036539	0.586406907	0.393579357
**RBFOX2**	0.022325305	0.335399488	0.577230597	0.474437605
**GCFC2**	0.00184449	0.262584678	0.540465552	0.580730618
**SF3B3**	0.072147369	0.025198193	0.267100847	1.5986306
**C1QBP**	-0.005186416	0.527907945	0.608241763	0.277441802
**RBM5**	-0.017996329	0.40879227	0.586406907	0.388497325
**MFAP1**	0.000833511	0.695445287	0.705427756	0.157737031
**ZMAT5**	-0.011644614	0.469411973	0.601289043	0.328445838
**ZRANB2**	-0.018327624	0.436911287	0.590921467	0.359606736
**PRPF4**	0.022158832	0.36507622	0.57798646	0.437616455
**DDX23**	0.021760787	0.508711441	0.601289043	0.293528495
**HNRNPF**	0.001263825	0.568871173	0.624874035	0.244986073
**WBP11**	0.02026731	0.411901196	0.586406907	0.385206947
**LSM1**	0.003828067	0.167541154	0.486791972	0.775878497
**RBM42**	-0.027112995	0.37176377	0.579316575	0.429732936
**UPF3B**	0.03154628	0.160469939	0.476850978	0.794606312
**CIRBP**	-0.075689357	0.016681892	0.20042237	1.777754694

**ZCRB1**	0.003505179	0.621578454	0.648657052	0.206504048
**YJU2**	0.001626537	0.651907857	0.667654424	0.185813784
**SON**	0.00049629	0.658969012	0.673259907	0.181135007
**CWC27**	-0.006504139	0.613183782	0.645136287	0.21240934
**HSPA8**	0.026049749	0.036783363	0.289139871	1.434348567
**RAVER2**	-0.007855357	0.56076352	0.621003717	0.251220246
**DHX15**	0.006661245	0.499292636	0.601289043	0.301644839
**SRPK3**	0.004062363	0.431142317	0.590921467	0.365379349
**PPIL4**	-0.008134238	0.563883092	0.621003717	0.248810928
**RNVU1-2A**	-0.00651372	0.776613348	0.78214741	0.109795149
**CPSF1**	0.014853985	0.379142339	0.58245055	0.421197715
**SYNCRIP**	0.030712225	0.327959851	0.577230597	0.48417932
**HNRNPD**	0.004340066	0.612230316	0.645136287	0.213085169
**PPP1R8**	0.002769853	0.513579747	0.601289043	0.289392111
**DHX35**	-0.0040715	0.585153251	0.62811387	0.232730378
**SETX**	-0.028395044	0.546240086	0.620927068	0.262616432
**SRSF11**	-0.03024519	0.286521604	0.558902963	0.542842626
**PCBP1**	-0.005292691	0.618867818	0.648657052	0.208402101
**RAVER1**	0.094039632	0.014177764	0.180585613	1.848392247
**SRPK1**	0.038137314	0.266930386	0.543635838	0.573601986
**DDX41**	-0.006979018	0.561796026	0.621003717	0.250421337
**U2AF1L4**	0.034000342	0.356164056	0.57798646	0.448349912
**SF3A3**	0.034089168	0.315439849	0.576493517	0.501083444
**METTL14**	0.014348234	0.223316468	0.518516996	0.651079249
**KHDRBS1**	-0.015242961	0.629968693	0.65307268	0.200681032
**PRPF19**	0.023414714	0.621340801	0.648657052	0.206670127
**CPSF4**	-0.01059227	0.507905732	0.601289043	0.294216886
**SNUPN**	0.039676537	0.028324646	0.285944046	1.547835508
**PCF11**	-0.022698922	0.506247762	0.601289043	0.295636884
**SNRPB**	-0.021871041	0.431673114	0.590921467	0.364845
**SCAF11**	-0.004107618	0.401626031	0.586406907	0.396178147
**HNRNPH3**	0.023266508	0.514496537	0.601289043	0.288617544
**SART3**	0.03215406	0.295652009	0.560381966	0.529219166
**GEMIN5**	0.016148461	0.429192365	0.590921467	0.367348012
**HNRNPA1**	0.012220953	0.286391466	0.558902963	0.543039927
**DDX1**	-0.004812519	0.709800819	0.716560827	0.148863504
**TRA2A**	6.60041E-05	0.418077609	0.586406907	0.378743091
**SMN2**	0.060297266	0.009884988	0.155230917	2.005023871
**COIL**	0.012329634	0.47694519	0.601289043	0.321531527
**RBM28**	-0.010722918	0.485146224	0.601289043	0.314127344
**DHX8**	0.01821133	0.483508705	0.601289043	0.315595703
**SMNDC1**	-0.048378542	0.10558946	0.42228249	0.97637943
**HNRNPUL1**	-0.034213615	0.322585885	0.577230597	0.49135464
**CSTF3**	0.004452722	0.553842309	0.621003717	0.25661387
**SMC1A**	0.053208022	0.154584639	0.471538755	0.810833664
**PRPF8**	-0.007020015	0.439854835	0.590921467	0.35669063
**POLR2J**	0.163095808	0.004961507	0.123745827	2.304386373
**CWC15**	-0.012959155	0.387414421	0.582495441	0.411824218

**USP4**	-0.032223759	0.414653021	0.586406907	0.382315166
**LARP7**	-0.02448947	0.345923874	0.577230597	0.461019464
**NCBP1**	0.026990693	0.556630741	0.621003717	0.254432812
**ELAVL1**	-0.029626657	0.326154816	0.577230597	0.486576204
**U2AF1**	0.002946981	0.482612952	0.601289043	0.316401027
**WTAP**	0.048236862	0.101981713	0.42228249	0.991477699
**PPIL1**	0.046925191	0.148602197	0.470031869	0.82797477
**SRSF5**	-0.000612596	0.637445697	0.656665272	0.195556806
**POLR2G**	0.014158398	0.516579535	0.601289043	0.286862803
**DDX5**	-0.002189945	0.638080406	0.656665272	0.195124592
**RALY**	-0.014209078	0.510446789	0.601289043	0.292049524
**DHX38**	0.029936183	0.466234658	0.601289043	0.331395446
**SRSF4**	0.016315519	0.59764827	0.635094903	0.223554333
**MYEF2**	-0.020402744	0.047393659	0.318966848	1.32427976
**POLR2C**	0.040045315	0.211679495	0.518516996	0.674321209
**SNU13**	0.016078517	0.704605311	0.713013489	0.152054087
**PPIE**	0.020203188	0.517134196	0.601289043	0.286396743
**SF3B2**	-0.003440472	0.520455374	0.601289043	0.283616502
**SNIP1**	0.020311864	0.625959609	0.650507044	0.20345369
**CWC25**	-0.001981747	0.622649576	0.648657052	0.205756303
**NUP98**	-0.038435672	0.286644754	0.558902963	0.542656002
**CELF6**	0.019689602	0.092384645	0.42228249	1.034400205
**POLR2B**	-0.02754632	0.365440879	0.57798646	0.437182874
**LSM4**	0.025796963	0.45333368	0.600667126	0.343582014
**DNAJC8**	0.047927812	0.204326145	0.518516996	0.689676058
**PTBP1**	0.015278159	0.581144257	0.62811387	0.23571605
**PLRG1**	0.026755745	0.477002085	0.601289043	0.321479723
**HNRNPA1P60**	-0.001613228	0.831102559	0.834563712	0.08034538
**RBM17**	0.034090012	0.439281489	0.590921467	0.357257098
**SRPK2**	-0.026724441	0.206607241	0.518516996	0.684854462
**EFTUD2**	0.056168057	0.095933499	0.42228249	1.018029714
**ESS2**	-0.018311361	0.465748833	0.601289043	0.331848225
**SNRPC**	0.028441966	0.477210811	0.601289043	0.321289726
**POLR2I**	0.036063417	0.373003361	0.579316575	0.428287255
**AAR2**	0.019570065	0.612060143	0.645136287	0.2132059
**RNVU1-17**	-0.001000644	0.998727423	0.998727423	0.000553025
**RNU5E-1**	0.565544669	0.000896193	0.042220635	3.04759859
**DGCR14**	0.13615249	0.027301427	0.282336712	1.563814647
**RNVU1-6**	0.06488026	0.096996381	0.42228249	1.01324447
**SKIV2L2**	-0.079649743	0.139345627	0.454481122	0.855906656
**RNVU1-19**	0.036350317	0.386126487	0.582495441	0.413270406
**HNRNPA1L2**	0.023792865	0.503111054	0.601289043	0.298336141
**C7orf55-LUC7L2**	-0.004359538	0.832595401	0.834563712	0.079565992

Status
Up
Up
Stable
Stable
Stable
Stable
Stable
Stable
Stable
Stable
Stable
Stable
Stable
Stable
Stable
Up
Stable
Stable
Stable
Stable
Stable
Stable
Stable
Stable
Stable
Stable
Stable
Stable
Stable
Stable
Stable
Stable
Stable
Stable
Stable
Stable
Stable
Stable
Stable
Up
Stable
Stable
Stable
Stable
Up

Stable
Stable
Stable
Stable
Stable
Stable
Stable
Stable
Stable
Stable
Stable
Stable
Stable
Stable
Up
Stable
Stable
Stable
Stable
Stable
Stable
Stable
Stable
Stable
Stable
Stable
Stable
Stable
Stable
Stable
Stable
Stable
Stable
Stable
Stable
Stable
Stable
Stable
Stable
Stable
Stable
Stable
Stable
Stable
Stable
Stable
Stable
Stable

Stable
Stable
Stable
Stable
Stable
Stable
Stable
Stable
Stable
Stable
Stable
Stable
Stable
Stable
Stable
Stable
Stable
Stable
Stable
Stable
Stable
Stable
Stable
Stable
Stable
Stable
Stable
Stable
Stable
Stable
Stable
Stable
Stable
Stable
Stable
Stable
Stable
Stable
Stable
Stable
Stable
Stable
Stable
Stable
Stable
Stable
Stable
Stable

Stable
Stable
Stable
Stable
Stable
Stable
Up
Stable
Stable
Stable
Stable
Stable
Stable
Stable
Stable
Stable
Stable
Stable
Stable
Up
Stable
Stable
Stable
Stable
Stable
Stable
Stable
Stable
Stable
Stable
Stable
Stable
Stable
Stable
Stable
Stable
Stable
Stable
Stable
Stable
Stable
Stable
Stable
Stable
Stable
Stable
Stable
Stable

Stable
Stable
Stable
Stable
Stable
Stable
Stable
Stable
Stable
Stable
Stable
Stable
Stable
Stable
Stable
Stable
Stable
Stable
Stable
Stable
Stable
Stable
Stable
Stable
Stable
Stable
Stable
Stable
Stable
Stable
Stable
Stable
Stable
Stable
Stable
Stable
Stable
Stable
Stable
Stable
Stable
Stable
Down
Stable
Stable
Stable
Stable
Stable

Stable
Stable
Stable
Stable
Stable
Up
Stable
Up
Stable
Stable
Stable
Stable
Stable
Stable
Stable
Stable
Stable
Stable
Stable
Stable
Stable
Stable
Stable
Stable
Stable
Stable
Stable
Stable
Stable
Stable
Stable
Stable
Stable
Stable
Stable
Stable
Stable
Stable
Stable
Stable
Stable
Stable
Stable
Stable
Stable
Stable
Stable
Stable

Stable
Stable
Stable
Stable
Stable
Stable
Stable
Stable
Stable
Stable
Stable
Stable
Stable
Stable
Stable
Stable
Stable
Stable
Stable
Stable
Stable
Stable
Stable
Stable
Stable
Stable
Stable
Stable
Stable
Stable
Stable
Stable
Stable
Stable
Stable
Stable
Stable
Stable
Stable
Stable
Stable
Stable
Stable
Stable
Stable
Stable
Stable
Stable

Stable
Stable
Stable
Stable
Stable
Stable
Stable
Stable
Stable
Stable
Stable
Stable
Stable
Stable
Stable
Stable
Stable
Stable
Stable
Stable
Stable
Stable
Stable
Stable
Stable
Stable
Stable
Stable
Stable
Stable
Stable
Stable
Stable
Stable
Stable
Stable
Stable
Stable
Stable
Stable
Stable
Stable
Stable
Stable
Stable
Stable
Up
Stable

Stable
Stable
Stable
Stable
Stable
Stable
Stable
Stable
Stable
Stable
Stable
Stable
Stable
Stable
Stable
Stable
Stable
Stable
Stable
Stable
Stable
Stable
Stable
Stable
Stable
Stable
Stable
Stable
Stable
Stable
Stable
Stable
Stable
Stable
Stable
Stable
Up
Up
Stable
Stable
Stable
Stable
Stable

Supplementary Table S5: Antibodies used in study

Antibody	Species	Supplier	Applications	Catalogue numb	WB Dilution	RRID
AR (441)	Mouse	Agilent Dako	WB, IF	M3562	1 in 1000	AB_3572260
AR (441)	Mouse	BD Biosciences	WB	554225	1 in 1000	AB_395316
AR-V7	Rabbit	RevMab Biosciences	WB	31-1109-00	1 in 500	AB_2716436
